# Structural behavior of reinforced concrete beams with longitudinal voids incorporating embedded steel tubes

**DOI:** 10.1038/s41598-026-59579-4

**Published:** 2026-07-09

**Authors:** Sabry Fayed, Mohamed Ghalla, Yahia Iskander, Rabeea W. Bazuhair, Yahya M. Bin Mahfouz, Saad A. Yehia

**Affiliations:** 1 Department of Civil Engineering, Faculty of Engineering, Kafrelsheikh University, Kafr El-Sheikh, Egypt; 2 Department of Civil Engineering, College of Engineering and Architecture, Umm Al-Qura University, Makkah, Saudi Arabia; 3 Civil Engineering Department, Higher Institute of Engineering and Technology, Kafr El-Sheikh, Egypt

**Keywords:** Reinforced concrete beams, Voids, Compression zone, Steel tubes, Failures, Ultimate load, Engineering, Materials science

## Abstract

The integration of utility services in modern buildings often requires longitudinal openings within reinforced concrete (RC) beams. However, these voids, particularly in the compression zone, significantly reduce structural capacity and ductility by decreasing the effective concrete area and inducing stress concentrations. This study investigates the structural behavior of RC beams with longitudinal voids incorporating embedded steel tubes as internal composite reinforcement. An experimental program comprising eleven RC beams tested under four-point bending was conducted and validated using three-dimensional nonlinear finite element analysis. The study examined the effects of void geometry, tube shape, tube orientation, and reinforcement ratio. Results showed that introducing an unreinforced void reduced the ultimate load capacity by 27.5% and caused substantial losses in stiffness and energy absorption compared to the solid beam. In contrast, embedded steel tubes significantly enhanced structural performance through composite action. The best rectangular double-tube configuration increased the ultimate load capacity by 150% relative to the unreinforced void beam, while the circular double-tube system achieved an improvement of 180%. Circular tubes exhibited slightly superior performance due to more uniform stress distribution, whereas horizontally oriented rectangular tubes outperformed vertical ones because of their higher moment of inertia. The developed finite element model accurately predicted the experimental behavior and failure modes. The findings demonstrate that embedded steel tubes provide an efficient and practical solution for improving the performance of RC beams with service-integrated longitudinal voids.

## Introduction

**Fig. 1:**
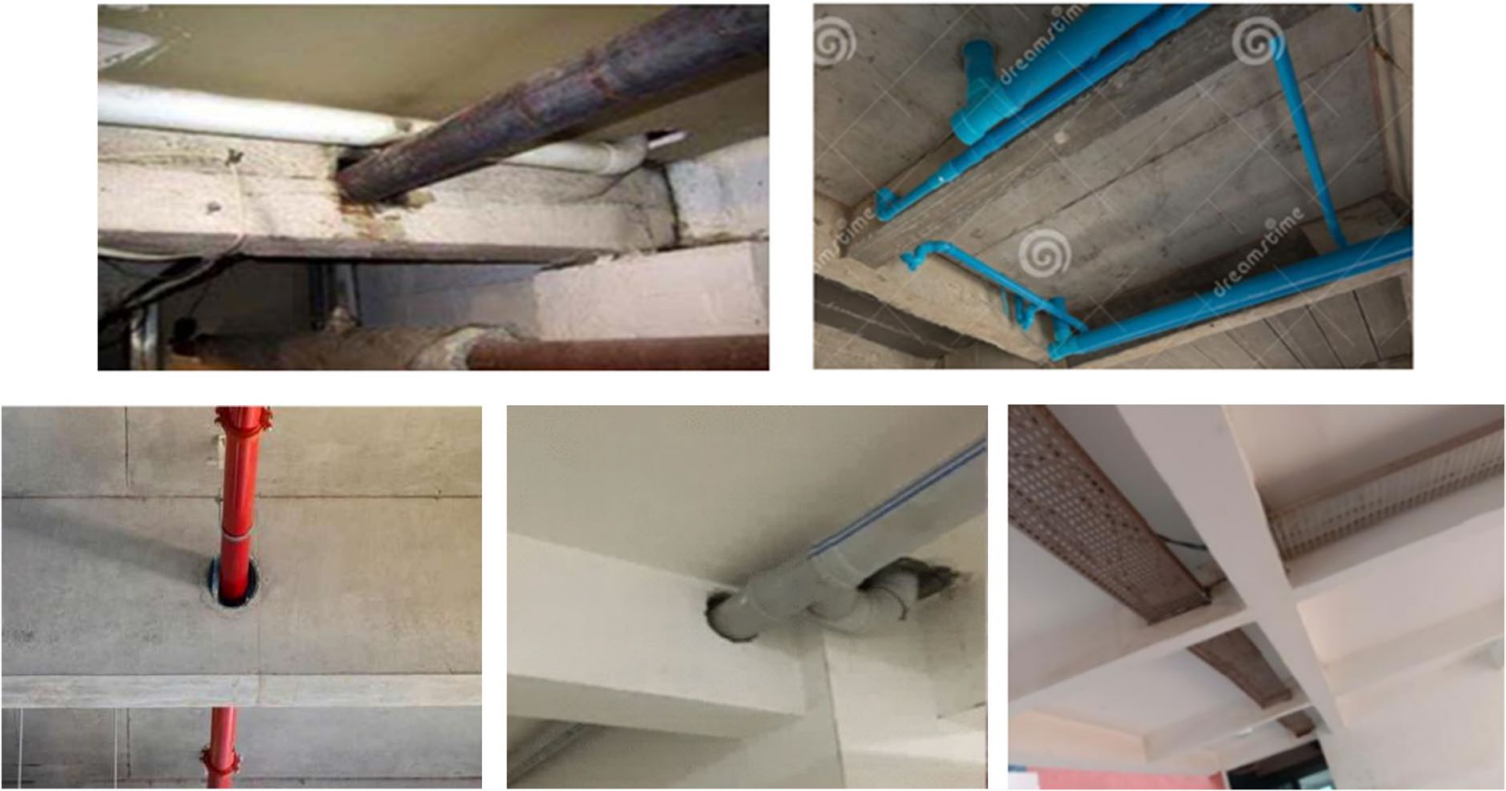
RC beams with web openings.

Reinforced concrete (RC) beams are among the most essential structural elements in buildings and infrastructure systems, as they primarily resist flexural and shear forces while safely transferring loads to supporting members. The structural behavior of RC beams depends on several factors, including cross-sectional geometry, reinforcement arrangement, material properties, and loading conditions^1–5^. Conventional solid RC beams generally provide adequate strength and stiffness. However, in high-rise buildings, vital utility services such as water supply systems, gas pipelines, electrical conduits, and telecommunication networks are often installed beneath reinforced concrete beams and concealed by suspended ceilings. Although this arrangement is functional, it frequently results in inefficient utilization of vertical space and increased story height, leading to greater material consumption and higher construction costs. Consequently, modern structural systems increasingly incorporate openings within floor beams to facilitate the direct passage of pipes and ducts through the beam depth (Fig. 1). This approach reduces headroom requirements while improving both structural and economic efficiency^6,7^.

The incorporation of openings in reinforced concrete (RC) beams has raised considerable concerns regarding structural integrity and serviceability performance, as such discontinuities complicate the conventional load-transfer mechanism of the beam^8^. Abrupt reductions in the cross-sectional area generate significant stress concentrations, particularly around the corners of openings, which may initiate excessive cracking and adversely affect both structural durability and aesthetic quality^9,10^. Previous studies investigating the flexural and shear behavior of RC beams with web opening have demonstrated that their structural response is highly dependent on the size, shape, and location of the openings^11–14^. For instance, Hassan et al.^15^ investigated the shear behavior of strengthened RC T-beams with horizontal and vertical web openings. Experimental results from thirteen beams showed that openings reduced shear capacity, particularly vertical openings. Moreover, the introduction of longitudinal holes modifies the internal stress distribution and reduces sectional stiffness, potentially resulting in premature crack propagation and a reduction in ultimate load-carrying capacity compared with conventional solid beams^16–19^. For instance, in a study by Balaji and Vetturayasudharsanan^20^, the influence of varying the number of circular openings along the beam’s length, while keeping the total void area constant, was explored. Their findings revealed that beams with a single large opening (63.5 mm) exhibited superior flexural capacity compared to those with two smaller openings (31.75 mm each), suggesting a potential design optimization for RC beams. In a recent experimental study, Elamary et al.^21^ conducted an experimental investigation to assess the impact of longitudinal voids on the flexural capacity of hollow RC beams. The results showed that failure load only slightly decreased when voids made up less than 10% of the cross-sectional area. Additionally, a numerical analysis explored the influence of transverse outlet positions—near supports or mid-span—on the beams’ overall structural performance. Murugesan and Narayanan^17^ investigated the flexural behavior of simply supported hollow RC beams containing longitudinal circular voids with varying diameters and locations. Experimental testing of twelve hollow beams showed flexural failure in all specimens. The study developed theoretical models accurately predicting cracking and ultimate loads while clarifying the influence of void size and position. Hassan et al.^22^ investigated RC beams containing longitudinal PVC pipe voids in the tension zone under four-point bending. Experimental results showed that small void diameters had negligible influence on strength and stiffness, whereas larger voids caused brittle shear failure.

In their study, Manikandan et al.^23^ explored the effect of longitudinal voids on the structural behavior of RC beams. The results showed that reducing concrete in the tensile zone by 25% did not significantly affect flexural capacity, deflection, or strain distribution. Hollow beams constructed from high-strength concrete ($\:{f}_{c}$ = 63–73 MPa) and featuring cross-sectional reductions of up to 44.4% performed similarly to solid beams in terms of toughness and load resistance^24^. The crack patterns and failure mechanisms were also comparable between both types^16–19^. Moreover, the superior ductility material^25^ exhibited by hollow engineered cementitious composite (ECC) beams with 60 mm and 80 mm openings, in comparison to solid beams, positions them as viable candidates for energy-dissipating applications^26^. Additionally, in their study, Alnuaimi et al.^27^ tested 14 beams and demonstrated that the torsion-to-bending moment ratio (0.19–2.62) plays a significant role in influencing the failure loads of both hollow and solid beams. Furthermore, the study also examined the impact of concrete compressive strength ($\:{f}_{c}$ = 46.2–96.7 MPa) and torsional reinforcement ratio (0.30–2.68%) on the cracking behavior of the beams^28^. Consequently, considerable research efforts have focused on developing strengthening techniques capable of restoring or enhancing the structural performance of such beams.

**Fig. 2:**
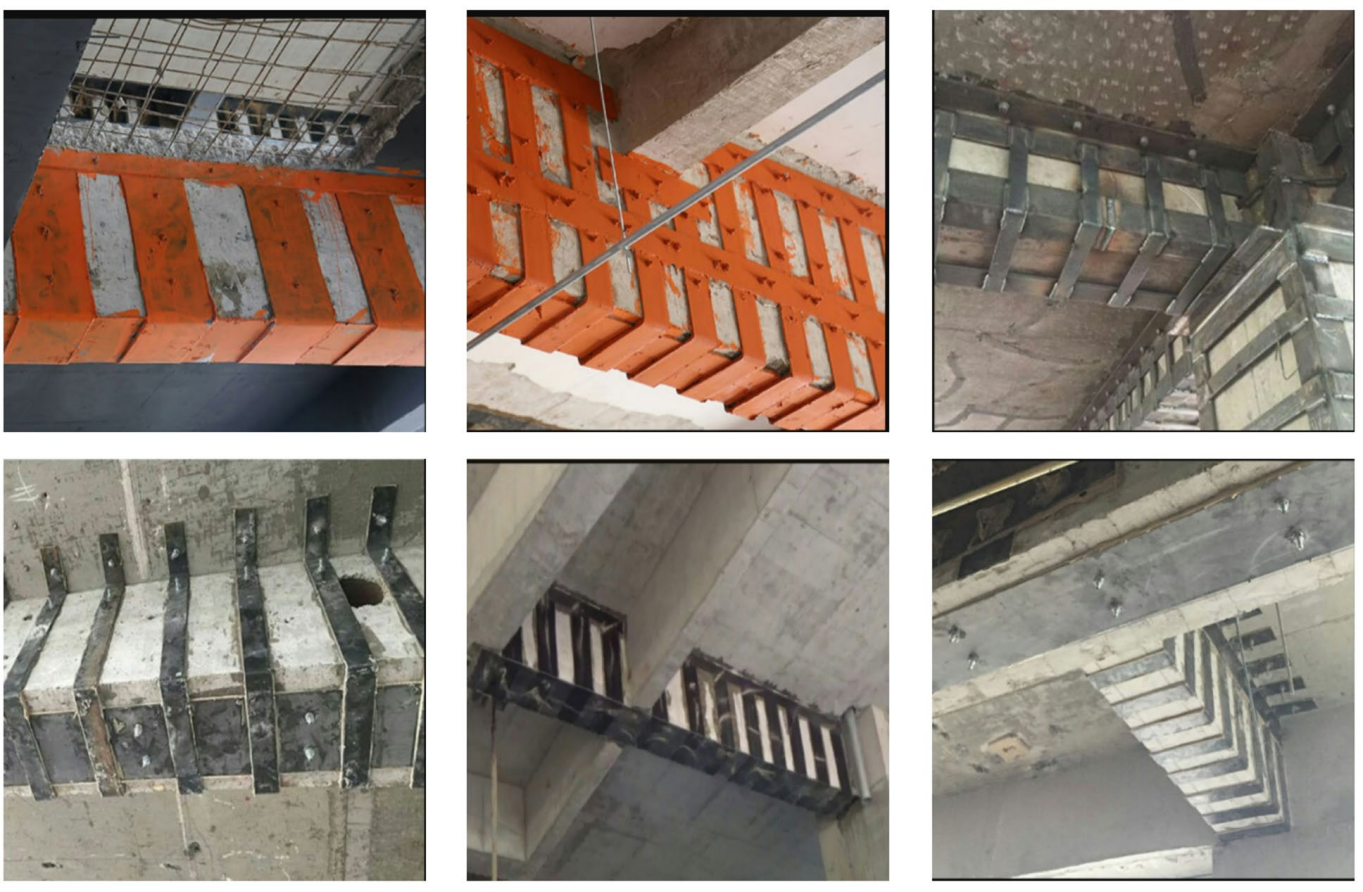
Strengthening RC beams with steel plates.

Several strengthening and rehabilitation methods have been proposed for RC beams with openings, including externally bonded fiber-reinforced polymer (FRP) sheets^29,30^, ECC jackets^31–37^, and steel plates^38–40^. FRP-based techniques have shown effectiveness in improving strength and crack control due to their high strength-to-weight ratio and corrosion resistance^41,42^. However, these methods may suffer from debonding problems, brittle failure modes, and relatively high material costs^43,44^. Steel-based strengthening systems, on the other hand, provide high ductility, improved energy absorption, ease of installation, and reliable confinement efficiency, making them attractive for strengthening RC structural members (Fig. 2). Consequently, several researchers have explored the use of steel plates and steel-based strengthening techniques to enhance the structural behavior of RC beams containing openings under different loading and geometric conditions. For instance, Hassan and Muhammad^45^ investigated galvanized steel plates for strengthening RC beams with drilled shear openings as a cost-effective alternative to FRP. Experimental results on seven beams showed shear capacity reductions up to 59.75% due to openings. Strengthening significantly improved capacity, enhancing ductility, altering failure modes, and enabling analytical prediction of shear resistance. In another study, Abdul-Razzaq and Abdul-Kareem^46^ investigated steel plate strengthening of RC T-beams with flange openings of various shapes and sizes. Unstrengthened openings reduced capacity up to 42% and increased deflection. Steel plates partially restored strength, with gains up to 44%, though performance was influenced by plate buckling under compressive stresses in thirteen full-scale beams. Allam^47^ experimentally investigated nine RC beams with shear-zone openings to evaluate internal and external strengthening effectiveness. Steel plates and CFRP sheets were applied with different configurations. Results showed that steel plates outperformed CFRP, restoring full shear capacity and changing failure from shear to flexure, with improved deflection, cracking, and strength.

Despite the increasing interest in strengthening RC beams using steel-based elements^48^, limited studies have examined the effectiveness of embedded steel tubes as internal composite reinforcement in beams with longitudinal voids. Moreover, their influence on the overall structural response of RC beams remains insufficiently explored. In particular, the behavior of RC beams containing compression-zone voids, with or without internal reinforcement, remains insufficiently explored. These beams typically exhibit brittle failure governed by premature concrete crushing before yielding tensile steel, unlike under-reinforced beams where ductile steel yielding governs failure.

To address these limitations and establish the feasibility of using embedded steel tubes as an internal strengthening technique, it is important to consider the well-documented behavior of concrete-filled steel tube (CFST) systems under different loading conditions^49–51^. Extensive research has been conducted on the compressive and flexural behavior of concrete-filled steel tube (CFST) members, demonstrating significant improvements in strength and structural efficiency^52,53^. Concrete-encased CFST members have gained widespread application in high-rise buildings and bridge structures owing to their cost-effectiveness and superior structural performance, including enhanced confinement of the core concrete, higher stiffness and load-carrying capacity, improved ductility and durability, reduced sectional dimensions, better fire resistance, prevention of local buckling and steel tube corrosion, and easier connection details with reinforced concrete or steel members^54,55^. Although numerous investigations have focused on the axial compressive behavior of CFST and reinforced concrete members, limited attention has been devoted to their flexural and shear responses^55–57^. Experimental and analytical studies by An et al.^58^ and Han et al.^54^ examined concrete-encased CFST members with the steel tube positioned at different locations within the section, including the tension and compression zones. However, because concrete possesses relatively low tensile strength, cracking rapidly develops in the tension region under bending, causing tensile stresses to transfer mainly to the steel reinforcement. Consequently, the compressive capacity of the concrete section is not fully utilized, leading to reduced confinement efficiency, stiffness, strength, and ductility of the CFST system. Previous studies also reported that the flexural stiffness of conventional CFST members becomes close to that of hollow steel tubes after concrete cracking. In contrast, placing the entire CFST section within the compression zone significantly enhances concrete confinement and enables more effective utilization of the concrete compressive strength^54,58^. Therefore, the present study adopts a sectional configuration in which the entire CFST is located within the compression zone to maximize structural efficiency under flexural loading.

## Research significance

This study presents a significant contribution to the field of structural engineering by introducing embedded steel tubes as an innovative internal strengthening system for reinforced concrete (RC) beams containing longitudinal voids in the compression zone. Unlike conventional strengthening techniques that rely on externally bonded materials such as FRP sheets or steel plates, the proposed system integrates steel tubes directly within the beam section, enabling efficient composite action and improved structural integrity. The research addresses a critical gap in the literature concerning the behavior of hollow RC beams with compression-zone voids, particularly regarding their load-carrying mechanism, stiffness degradation, ductility, and failure characteristics. Through an extensive experimental and numerical investigation, the study demonstrates that embedded steel tubes can substantially restore and enhance the structural performance of voided beams, achieving significant improvements in ultimate load capacity, stiffness, and energy absorption. Furthermore, the study provides a comprehensive evaluation of key parameters including void geometry, tube shape, tube orientation, and reinforcement ratio, offering valuable design insights for practical engineering applications. The validated three-dimensional nonlinear finite element model also provides a reliable analytical tool for predicting the behavior of such composite systems, which can support future optimization and large-scale implementation. The proposed technique contributes to the development of more sustainable and economically efficient structural systems by enabling the integration of utility services within beam depths while minimizing increases in story height and material consumption. Therefore, the findings of this research are highly relevant to advanced structural design, strengthening technologies, and modern construction practices.

## Experimental program

This section describes the experimental program undertaken to investigate the structural behavior of concrete beams with internal voids and embedded steel tubes. It details the materials used, the design and fabrication of eleven beam specimens, and the four-point bending test setup employed to evaluate their mechanical performance.

### Used materials

#### Normal concrete

Normal concrete (NC) was used throughout the study and consisted of 42.5-grade Portland cement, natural sand, graded crushed basalt dolomite, water, and a superplasticizer. The materials were selected to ensure adequate workability and mechanical performance. The concrete was prepared following standard laboratory procedures to achieve a homogeneous mix. Sieve analysis^59^ was conducted on the aggregates and verified using broken concrete specimens after testing to confirm proper aggregate gradation and uniform distribution within the concrete matrix. The mix design followed a ratio of 1:2.17:4.3 for cement, fine aggregate, and coarse aggregate, respectively, with a water-to-cement ratio of 0.50. The detailed mix proportions were 300 kg/m³ of cement, 150 kg/m³ of water, 650 kg/m³ of fine aggregate, 1290 kg/m³ of coarse aggregate, and 12 kg/m³ of superplasticizer (Table 1). To evaluate the mechanical behavior of the developed NC mix, compressive and splitting tensile strength tests were performed on six specimens. Three 150 mm cubes and three 150 × 300 mm cylinders were cast from the same mix and cured for 28 days. The compressive strength tests followed the Egyptian Code of Practice (ECP)^60^, yielding an average of 25 MPa. The splitting tensile strength, determined from the cylindrical specimens, recorded an average value of 1.85 MPa.

**Table 1:** Material proportions of the concrete mix utilized.

Concrete type	Cement ($\:kg/{m}^{3}$)	Water $\:(kg/{m}^{3})$	Fine aggregate ($\:kg/{m}^{3}$)	Coarse aggregate ($\:kg/{m}^{3}$)	Super plasticizer ($\:kg/{m}^{3}$)	Water/cement (%)
Normal concrete	300	150	650	1290	12	0.50

#### Steel reinforcement bars

In accordance with the provisions of the Egyptian Code of Practice (ECP)^60^, uniaxial tensile tests were performed to evaluate the mechanical properties of the reinforcing steel used for both longitudinal and transverse elements. The Ø16 mm bars exhibited a yield strength of 365 MPa and an ultimate tensile strength of 524 MPa, while the Ø8 mm bars recorded corresponding values of 256 MPa and 410 MPa. According to the ECP^60^ requirements for reinforcing steel, the specified yield strengths of both bar diameters fall within the acceptable ranges defined by the code. These ranges correspond to mild steel grade (240/350) and high-tensile grade (360/520) reinforcement commonly used in structural applications. A comprehensive summary of the test results is provided in Table 2.

**Table 2:** Properties of reinforcing rebars.

Bar	Use	Poisson’s ratio	Elastic modulus (GPa)	Yield stress (MPa)	Ultimate stress (MPa)
8 mm	Stirrups	0.3	202	256	410
16 mm	Longitudinal	0.3	202	365	524

#### Rectangular and circular tubular steel sections

Rectangular Tubular Steel Sections (RTs) and Circular Tubular Steel Sections (CTs) are widely used in structural applications due to their favorable mechanical performance, high strength-to-weight ratio, and geometric efficiency. Both section types used in this study were fabricated from structural steel conforming to Egyptian code of practice for steel construction and bridges^61^. Tension tests were carried out on samples taken from RT and CT to evaluate their yield and ultimate tensile strengths. The average nominal yield and ultimate tensile strengths of RT and CT were 360 MPa and 520 MPa, respectively. The RTs specimens featured sharp-cornered hollow profiles with uniform wall thickness, offering increased resistance to local buckling under axial and flexural loads due to their flat surfaces and higher section modulus about one axis. Conversely, CTs elements, characterized by a uniform circular cross-section, exhibited isotropic behavior in the transverse plane, making them particularly effective under torsional loading and multidirectional stress states. Both section types demonstrated elastic–plastic behavior with negligible strain hardening.

### Test specimens

**Fig. 3:**
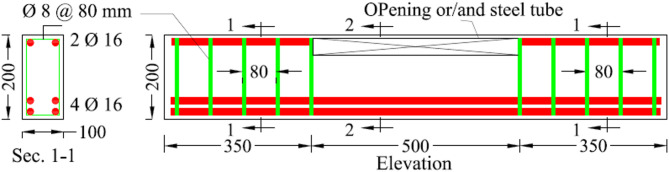
Layout of longitudinal section of all beams (dimensions in mm).

**Fig. 4:**
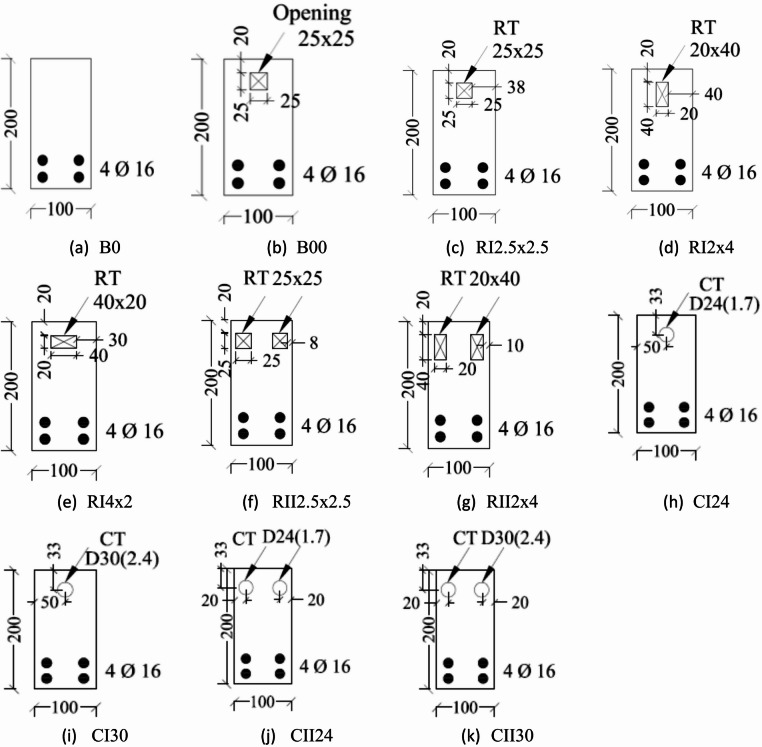
Configurations of openings reinforcement at compression zone (section 2-2) (dimensions in mm).

In this study, eleven RC beams incorporating various internal configurations were fabricated to investigate the effects of voids and embedded steel tubes on the structural behavior of RC beams. The detailed specifications of all specimens are presented in Figs. 3 and 4 and summarized in Table 3. The longitudinal openings introduced in the compression zone were intended to simulate practical service ducts and utility passages commonly required in modern buildings for electrical conduits, plumbing systems, ventilation ducts, and communication networks. Providing such openings within the beam depth can significantly improve spatial efficiency and reduce floor-to-floor height requirements by eliminating the need for suspended ceilings or external service routing. However, these openings reduce the effective concrete area and alter the internal stress distribution, which may adversely affect the load-carrying capacity, stiffness, and ductility of the beam. Therefore, this study investigates the feasibility of restoring and enhancing the structural performance of hollow RC beams through the use of internally embedded steel tubular sections acting simultaneously as reinforcement and permanent formwork for the openings.

All beams shared identical geometric dimensions, measuring 100 mm in width, 200 mm in overall depth, and 1200 mm in length. Longitudinal reinforcement consisted of four 16 mm diameter steel bars in the tension zone and two 16 mm diameter bars in the compression zone, with the latter confined to 350 mm from each end of the beam. The parameters of these beams are: compression concrete strength fc’ was 25 MPa, steel yield value fy was 315 MPa, beam width b was 100 mm, beam depth d was170 mm and cross-sectional area of reinforcement As was 804 mm^2. The balanced area of reinforcement was found to be 388 mm^2. Actual and balanced reinforcement ratios were estimated to be 4.73% and 2.28%, respectively, hence this section is considered as over-reinforced type.

The central region between the loading points was intentionally left unreinforced in compression and without shear reinforcement. Shear reinforcement was provided in the form of 8 mm diameter stirrups spaced at 80 mm intervals, applied only within the 350 mm end regions, as illustrated in Fig. 3.

The specimen matrix, as presented in Table 3, includes control, void-only, and void-with-tube specimens featuring both rectangular and circular geometries. The control specimen, B0 (Fig. 4a), was a solid beam without any openings or embedded steel tubes. Specimen B00 (Fig. 4b) contained a single square void (25 × 25 mm), representing an opening-to-beam section ratio (β) of 3.13%, but no embedded tube. Beams in the RI and RII series were configured with rectangular voids and rectangular tubular steel sections (RTs). The RI-series beams (RI2.5 × 2.5 (Fig. 4c), RI2 × 4 (Fig. 4d), and RI4 × 2 (Fig. 4e)) had one or two voids and one embedded rectangular tube, with β values ranging from 3.13% to 4.00% and corresponding tube-to-beam section ratios (ρ) from 5.0% to 6.40%. The RII-series beams (RII2.5 × 2.5 (Fig. 4f) and RII2 × 4 (Fig. 4g)) included two voids and two embedded tubes, with increased β values of 6.25% and 8.00%, and ρ values of 10.0% and 12.80%, respectively.

In contrast, the CI and CII series incorporated circular voids and circular tubular steel sections (CTs). The CI-series beams (CI24 (Fig. 4h) and CI30 (Fig. 4i)) included a single circular void with diameters of 24 mm and 30 mm, corresponding to β values of 2.26% and 3.53%, respectively. These specimens were reinforced with one circular steel tube, resulting in ρ values of 3.84% and 8.48%. The CII-series beams (CII24 (Fig. 4j) and CII30 (Fig. 4k)) featured two circular voids and two corresponding tubes, with increased void areas (β = 4.52% and 7.07%) and higher tube-to-beam section ratios (ρ = 7.69% and 16.96%). Overall, the beams were designed to cover a wide range of void and tube configurations, enabling a systematic investigation of how the geometry, quantity, and reinforcement of internal voids influence the structural performance of RC beams.

It should be noted that each specimen configuration was tested once due to experimental limitations. Since each beam configuration was tested without replicate specimens, a statistical evaluation of experimental scatter and repeatability could not be performed. Nevertheless, the reliability and consistency of the experimental program were maintained through strict control of specimen fabrication, uniform material properties, standardized casting and curing procedures, calibrated instrumentation, and identical loading and boundary conditions for all tests. Furthermore, the experimental findings were corroborated through a validated three-dimensional nonlinear finite element model, which demonstrated close agreement with the measured structural responses, thereby enhancing confidence in the observed behavioral trends and overall conclusions.

### Preparing and casting specimens

**Fig. 5:**
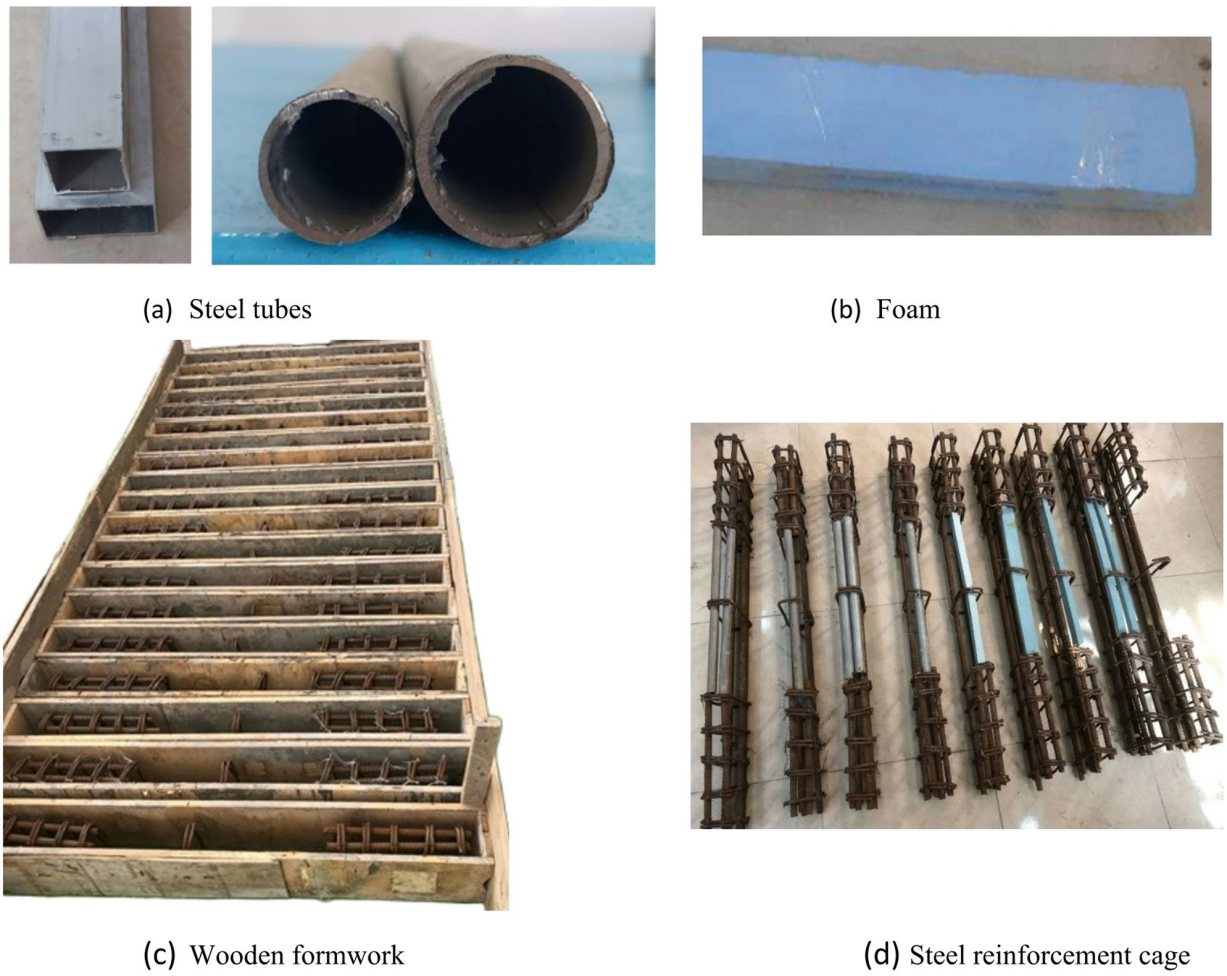
Overview of materials applied in specimen fabrication.

The fabrication of the eleven RC beams was conducted through a systematic three-stage process to ensure precision and uniformity across all specimens. In the first stage, steel tubes (Fig. 5a) were fabricated according to the specified geometric dimensions, as shown in Figs. 3 and 4. These tubes were carefully cut and shaped to fit precisely within the beam cross-section. Concurrently, compressed foam inserts were prepared and molded to form the internal voids in accordance with the required dimensions (Fig. 5b). At this stage, the wooden formwork (Fig. 5c) was meticulously constructed and aligned to match the exact beam dimensions, thereby facilitating accurate placement and support during concrete casting. The second stage focused on the assembly of the reinforcement cages (Fig. 5d). Steel reinforcement bars were fabricated and positioned according to the designed reinforcement layout. Proper alignment was ensured during assembly. Stirrups were then installed at the beam ends with uniform spacing. Special attention was devoted to securing the reinforcement cages, particularly in beams incorporating internal voids or embedded steel tubes, to prevent any displacement or misalignment during the subsequent casting process. Finally, in the third stage, concrete was cast using a normal concrete (NC) mix. Mechanical vibration was employed throughout the casting operation to ensure thorough compaction, eliminate entrapped air, and achieve full consolidation around the reinforcement and embedded components.

### Loading scheme

**Fig. 6:**
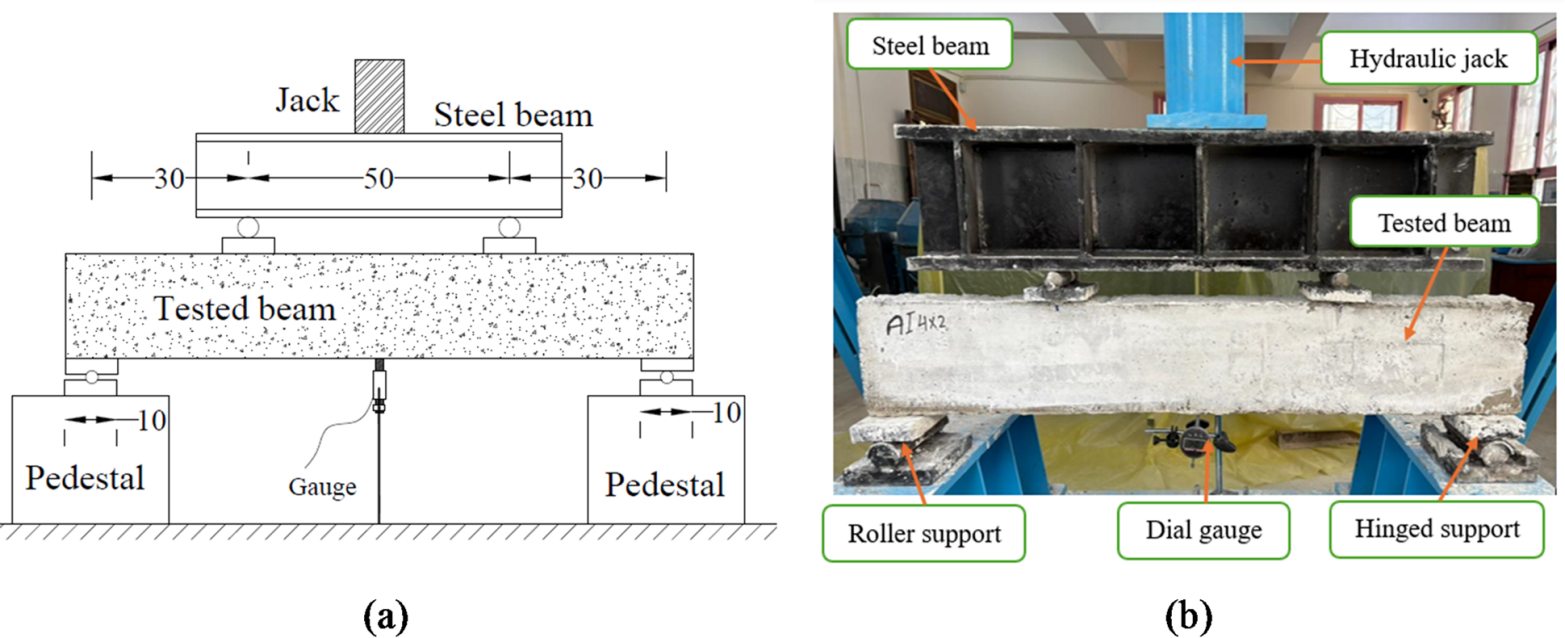
Test set up. (**a**) Schematic diagram, (dimensions in cm). (**b**) Actual test.

As illustrated in Fig. 6, all beam specimens were tested under four-point bending to evaluate their flexural behavior and failure characteristics. Prior to testing, the beam surfaces were coated with a white marking layer to facilitate crack observation and propagation monitoring during loading. The applied load was delivered through a 50-ton hydraulic jack mounted vertically on a rigid steel spreader beam, producing two equal concentrated loads positioned 300 mm from each support. This arrangement generated a constant bending moment region in the middle portion of the beam, where the openings and embedded steel tubes were located.

The beams were tested as simply supported members using a hinged support at one end and a roller support at the opposite end to permit free rotation and horizontal movement, thereby minimizing unintended restraint effects and secondary stresses. Mid-span deflections were continuously measured using a dial gauge positioned directly beneath the beam centerline.

Loading was applied incrementally in a quasi-static manner with careful manual control near the ultimate stage, enabling continuous monitoring of the structural response and allowing the post-peak behavior of some specimens to be captured prior to complete failure. This procedure facilitated the observation of the descending branches in the load–deflection curves of relatively brittle specimens without sudden unloading immediately after peak load attainment.

The tested specimens were designed and analyzed as flexural members rather than deep beams. According to commonly accepted classifications in reinforced concrete design codes (Egyptian code^60^), conventional beams are those whose span-to-effective depth ratio exceeds four. In the present study, the adopted loading configuration resulted in net span of 1100 mm and depth of 170 mm. So, span-to-effective depth ratio of the current beams is 6.8 which exceeds four. Ensuring that these beams primarily exhibited flexural behavior rather than deep beam action. Furthermore, the observed crack patterns and failure modes confirmed the predominance of flexural response within the constant moment region. Load and deflection data were recorded continuously throughout the testing process until specimen failure.

Although additional instrumentation such as strain gauges, multiple LVDTs, and quantitative crack-width measurements was not employed, the primary objective of the experimental program was to evaluate the global structural response of the specimens in terms of ultimate load capacity, deflection behavior, stiffness, energy absorption, crack propagation, and failure modes. The adopted instrumentation was therefore considered sufficient to achieve the intended comparative assessment among the investigated configurations. Furthermore, the experimentally observed failure mechanisms were supported by a validated nonlinear finite element model, which demonstrated good agreement with the measured responses and provided additional verification of the structural behavior and failure trends.

**Table 3:** Beams matrix.

Beams			Opening geometry				Tube geometry						
	Number (*N*)	Width b (mm)		Height h (mm)	Area A_o_ (mm^2^)	β (%)	Type	Number (*N*)	Width b (mm)	Height h (mm)	Thickness t (mm)	Area A_t_ (mm^2^)	ρ (%)
B0	0.0	0.0		0	0.0	0.0	N/A	0.0	0.0	0.0	N/A	0.0	0.0
B00	1.0	25.0		25.0	625.0	3.13	N/A	0.0	25.0	25.0	N/A	0.0	0.0
RI2.5 × 2.5	1.0	25.0		25.0	625.0	3.13	RT	1.0	25.0	25.0	1.60	1000.0	5.0
RI2 × 4	1.0	20.0		40.0	800.0	4.00	RT	1.0	20.0	40.0	1.60	1280.0	6.40
RI4 × 2	2.0	40.0		20.0	800.0	4.00	RT	1.0	40.0	20.0	1.60	1280.0	6.40
RII2.5 × 2.5	2.0	25.0		25.0	1250.0	6.25	RT	2.0	25.0	25.0	1.60	2000.0	10.0
RII2 × 4	2.0	20.0		40.0	1600.0	8.00	RT	2.0	20.0	40.0	1.60	2560.0	12.80
CI24	1.0	Dia. 24			452.16	2.26	CT	1.0	Dia. 24		1.70	768.67	3.84
CI30	1.0	Dia. 30			706.5	3.53	CT	1.0	Dia. 30		2.40	1695.60	8.48
CII24	2.0	Dia. 24			904.32	4.52	CT	2.0	Dia. 24		1.70	1537.34	7.69
CII30	2.0	Dia. 30			1413.0	7.07	CT	2.0	Dia. 30		2.40	3391.20	16.96

Where, RT is Rectangular tubular steel sections, CT is Circular tubular steel sections, Ao is cross-sectional area of openings, β is openings-to-beam section ratio, A_t_ is cross-sectional tubes, and ρ is tubes-to-beam section ratio area.

## Results and discussion

Table 4 presents a summary of the experimental results for twelve RC beams, systematically categorized into six groups based on distinct void characteristics and reinforcement configurations. The investigation focused on key performance indicators, including failure modes, load-carrying capacity, stiffness, and energy absorption of concrete beams^62^. This classification framework facilitates a comprehensive evaluation of the influence of void geometry and reinforcement detailing on the ductility and structural performance of RC beams.

### Failure patterns

**Fig. 7:**
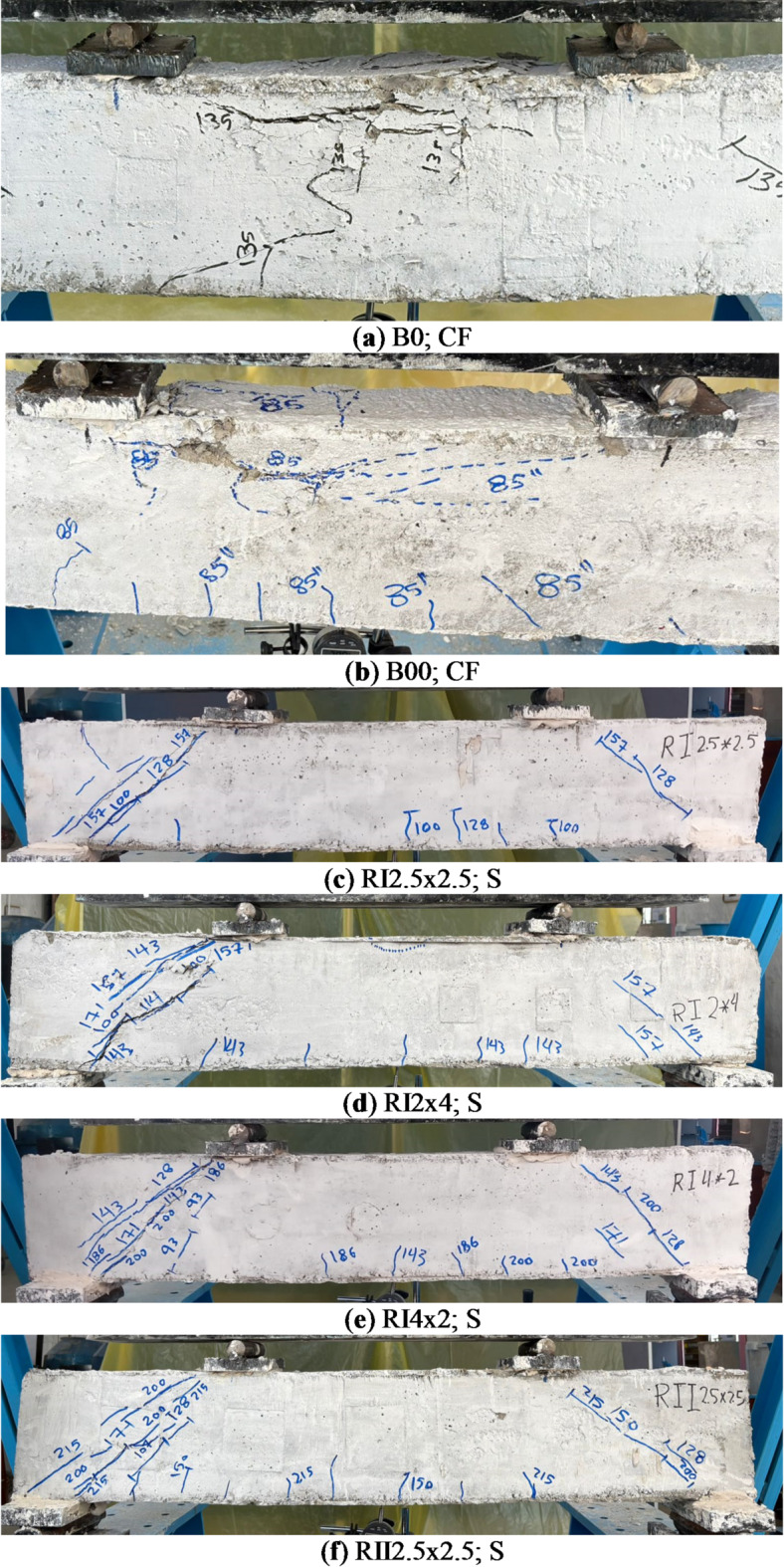

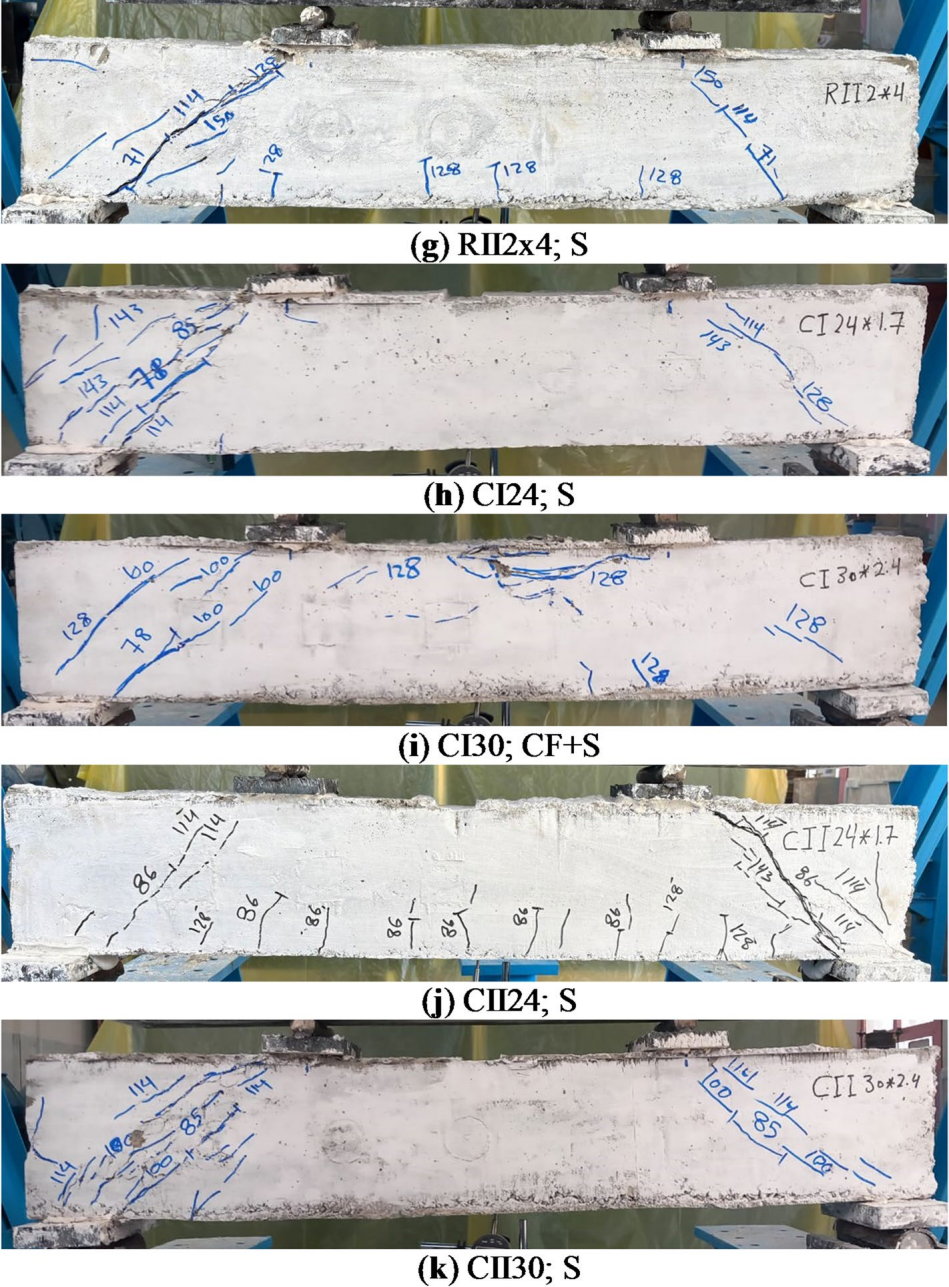
Crack patterns and failures of tested beams (CF is compression-flexure failure. S is shear failure).

Failure modes of the tested beams are shown in Fig. 7. Shear cracks were observed in all beams within the shear spans between the loading point and the supports. The first shear crack generally appeared at the early stages of loading. With increasing load, the crack width and length progressively increased, and additional shear cracks developed, propagating from the loading point toward the support regions.

The control beams (B0 and B00), shown in Fig. 7a, b, exhibited compression–flexure (CF) failure characterized by concrete crushing in the compression zone at midspan, including the unreinforced void region. This behavior is attributed to the relatively high reinforcement ratio in the tension zone. Although these beams sustained higher loads compared to beams with unreinforced voids, no compression-zone crushing was observed in the beams reinforced with embedded steel tubes, either circular or rectangular, around the voids.

For the reinforcement beams, the governing failure mode was predominantly shearing failure (S), as shown in Fig. 7c–h and j–k, despite the presence of internal steel tubes and the reduction in concrete area caused by the voids. Among these, beam CI30 (Fig. 7i) exhibited a combined compression–flexure and shear (CF + S) failure mode. Overall, shear cracks governed the response of all tube-reinforced specimens, indicating that although the embedded steel tubes enhanced flexural performance, shear failure remained the critical failure mechanism.

### Load versus deflection

**Fig. 8:**
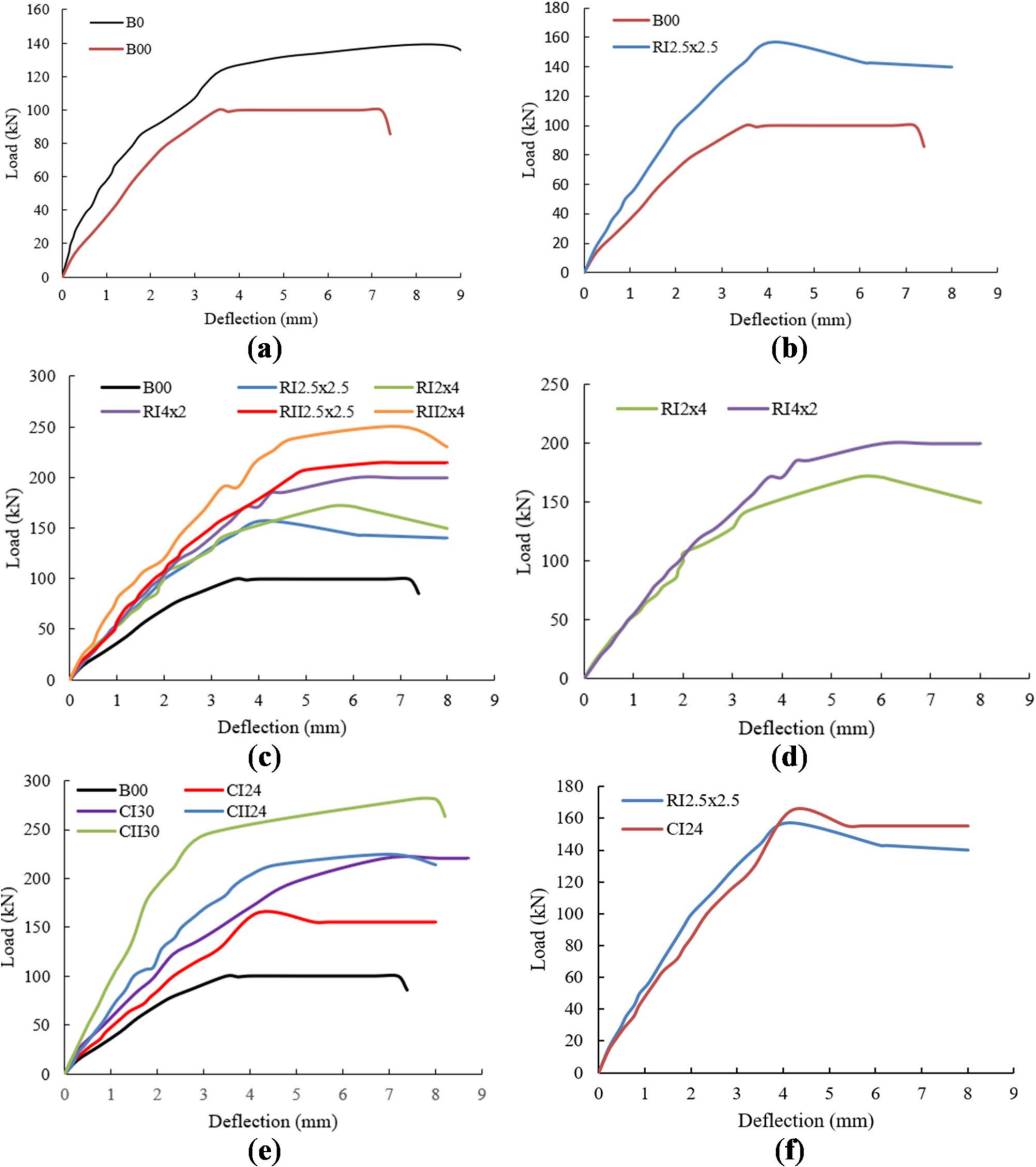
Load-deflection curves of all tested beams. (**a**) Effect of openings on P–δ curves of group G0. (**b**) Effect of using reinforcement on P–δ curves of group GI. (**c**) Effect of increasing rectangular tubes reinforcement ratio on P–δ curves of group GII. (**d**) Effect of tube orientation on P–δ curves of group GIII. (**e**) Effect of increasing circular tubes reinforcement ratio on P–δ curves of group GIV. (**f**) Effect of tube type on P–δ curves of group GV.

The monotonic load–deflection (P–δ) responses of all tested beams, as illustrated in Fig. 8a–f, exhibit three distinct behavioral phases. The initial phase is linear and elastic, extending up to the formation of the first visible cracks. This is followed by a nonlinear stage characterized by progressive crack propagation and damage accumulation, culminating at the peak load. Variations observed during this phase highlight the influence of internal void geometry and reinforcement detailing. The post-peak phase demonstrates either a plateau or gradual decline in load with increasing deflection, indicating structural softening. The observed energy dissipation capacity and failure modes are closely linked to the presence of voids and embedded steel tubes. Figure 8a compares the solid control beam (B0) with the unreinforced voided beam (B00). B0 exhibited a typical ductile response with an ultimate load of 138 kN and a deflection of 4.30 mm. In contrast, B00 showed a 27.5% reduction in ultimate load (100 kN) and an 18.6% reduction in deflection (3.50 mm), confirming that an unreinforced longitudinal void significantly impairs both strength and deformation capacity. Figure 8b demonstrates the effect of embedding a steel tube within the void. Beam RI2.5 × 2.5 (ρ = 5.0%) achieved an ultimate load of 157.1 kN and a deflection of 4.15 mm, corresponding to increases of 57.1% and 18.6%, respectively, compared to B00. The initial slope also became steeper, reflecting improved stiffness and delayed crack propagation due to composite action between the steel tube and surrounding concrete.

Figure 8c and e illustrate the influence of increasing the reinforcement ratio for rectangular and circular tubes, respectively. For rectangular tubes (Fig. 7c), increasing ρ from 5.0% (RI2.5 × 2.5) to 12.8% (RII2 × 4) raised the ultimate load from 157.1 kN to 250.1 kN (+ 59.2%) and deflection from 4.15 mm to 7.10 mm (+ 71.1%). Similarly, for circular tubes (Fig. 8e), increasing ρ from 3.84% (CI24) to 16.96% (CII30) increased the ultimate load from 165.1 kN to 280.0 kN (+ 69.6%) and deflection from 4.20 mm to 7.50 mm (+ 78.6%). These trends confirm that higher steel tube ratios proportionally enhance strength, stiffness, and ductility. Figure 8d compares the effect of rectangular tube orientation. Despite identical void and reinforcement ratios (β = 4.0%, ρ = 6.4%), the horizontally oriented tube in RI4 × 2 achieved an ultimate load of 200.4 kN and deflection of 6.10 mm, outperforming the vertically oriented RI2 × 4 (171.0 kN and 5.48 mm). This represents improvements of 17.2% in load and 11.3% in deflection, attributed to the higher moment of inertia and more efficient stress distribution of the horizontal configuration.

Figure 8f compares rectangular (RI2.5 × 2.5) and circular (CI24) tubes with similar reinforcement levels. CI24 exhibited a slightly higher ultimate load (165.1 kN vs. 157.1 kN, + 5.1%) and comparable deflection (4.20 mm vs. 4.15 mm), indicating that circular tubes provide marginally better performance due to smoother stress flow and reduced stress concentration around void corners. Overall, the load–deflection curves transitioned from brittle-like behavior in void-only beams to more ductile, resilient responses as steel tube reinforcement increased. The inclusion of steel tubes not only restored but often enhanced the structural performance beyond that of the solid control beam.

**Table 4:** Results of tested beams.

Parameter	Group	Beam	β (%)	ρ (%)	P_u_ (kN)	Change (%)	δ_u_ (mm)	Change (%)	k (kN/mm)	Change (%)	EA (kN.mm)	Change (%)
Presence of openings	G0	B0	0.00	0.00	138.00	0.00	4.30	0.00	60.40	0.00	1045.00	0.00
		B00	3.13	0.00	100.00	− 27.54	3.50	− 18.60	35.40	− 41.39	612.00	− 41.44
Using reinforcement	GI	B00	3.13	0.00	100.00	0.00	3.50	0.00	35.40	0.00	612.00	0.00
		RI2.5 × 2.5	3.13	5.00	157.10	57.10	4.15	18.57	51.10	44.35	956.00	56.21
Increasing rectangular tubes reinforcement ratio	GII	B00	3.13	0.00	100.00	0.00	3.50	0.00	35.40	0.00	612.00	0.00
		RI2.5 × 2.5	3.13	5.00	157.10	57.10	4.15	18.57	51.10	44.35	956.00	56.21
		RI2 × 4	4.00	6.40	171.00	71.00	5.48	56.57	52.20	47.46	1044.00	70.59
		RI4 × 2	4.00	6.40	200.40	100.40	6.10	74.29	52.30	47.74	1203.00	96.57
		RII2.5 × 2.5	6.25	10.00	214.00	114.00	6.50	85.71	56.40	59.32	1273.00	108.01
		RII2 × 4	8.00	12.80	250.10	150.10	7.10	102.86	61.50	73.73	1486.00	142.81
Tube orientation	GIII	RI2 × 4	4.00	6.40	171.00	0.00	5.48	0.00	52.20	0.00	1044.00	0.00
		RI4 × 2	4.00	6.40	200.40	17.19	6.10	11.31	52.30	0.19	1203.00	15.23
Increasing circular tubes reinforcement ratio	GIV	B00	3.13	0.00	100.00	0.00	3.50	0.00	35.40	0.00	612.00	0.00
		CI24	2.26	3.84	165.10	65.10	4.20	20.00	51.10	44.35	986.00	61.11
		CI30	3.53	8.48	220.90	120.90	6.90	97.14	53.00	49.72	1448.00	136.60
		CII24	4.52	7.69	225.00	125.00	7.02	100.57	57.10	61.30	1364.00	122.88
		CII30	7.07	16.96	280.00	180.00	7.50	114.29	95.10	168.64	1901.00	210.62
Tube type	GV	RI2.5 × 2.5	3.13	5.00	157.10	0.00	4.15	0.00	51.10	0.00	956.00	0.00
		CI24	2.26	3.84	165.10	5.09	4.20	1.20	51.10	0.00	986.00	3.14

### Overall characteristics

The P–δ response of each beam was analyzed to extract key structural performance parameters. The ultimate load capacity ($\:{P}_{u}$) was identified at the peak of the curve, while the corresponding ultimate deflection ($\:{\delta\:}_{u}$) was measured at the same stage. The elastic stiffness (k) was determined from the slope of the initial linear segment of the curve, while the energy absorption (EA) was calculated as the area under the P–δ curve up to $\:{P}_{u}$. These parameters, grouped according to the experimental classifications, are presented in Table 4.

#### Effect of the presence of openings (group G0)

Group G0 was established to assess the isolated effect of internal longitudinal openings on the structural behavior of concrete beams without any reinforcement. The results show that introducing an opening significantly reduced structural performance. Compared with the control beam, reductions of 27.5% in P_u_, 18.6% in δ_u_, over 41% in stiffness, and about 41% in energy absorption were observed. These reductions are mainly attributed to the loss of effective concrete area in the compression zone, which disturbed stress distribution and reduced flexural resistance. The opening also introduced a geometric discontinuity that increased stress concentration and accelerated crack initiation and propagation. The reduction in stiffness indicates a weakened elastic response, as the reduced moment of inertia led to larger deflections under identical loading. In addition, the lower ultimate deflection reflects a reduced deformation capacity, with earlier stiffness degradation and a more brittle post-cracking behavior. The absence of reinforcement around the opening further facilitated rapid crack propagation, limiting stress redistribution and post-cracking ductility. The decrease in energy absorption confirms a reduction in toughness and resistance to progressive damage under loading.

#### Effect of using reinforcement (group GI)

Group GI evaluates the influence of internal steel tube reinforcement on the structural behavior of voided RC beams. The results show that introducing the steel tube significantly enhanced both strength and deformation behavior. The $\:{P}_{u}$ increased by 57.10%, which is attributed to improved stress redistribution around the void and the composite action between concrete and steel, which delays crack initiation in the tensile zone and stabilizes the cracked section. More importantly, the deflection response shows a meaningful improvement in deformation capacity. The $\:{\delta\:}_{u}$ increased by 18.57%, indicating that the strengthened beam was able to sustain larger displacements before failure. This increase is not merely a numerical change but reflects a transition from a relatively brittle response in B00—where stiffness drops rapidly after cracking—to a more gradual and stable post-cracking behavior in RI2.5 × 2.5. The steel tube contributes to maintaining load transfer after cracking by bridging internal discontinuities and delaying rapid stiffness degradation, thereby enhancing ductility and allowing more energy to be dissipated through controlled cracking rather than sudden failure. The stiffness increased by 44.35%, reflecting the role of the steel tube in enhancing the effective flexural rigidity of the weakened section. In addition, energy absorption improved by 56.21%, confirming a substantial gain in structural toughness and the ability to sustain higher loads over a wider deformation range.

#### Effect of increasing rectangular tubes reinforcement ratio (group GII)

**Fig. 9:**
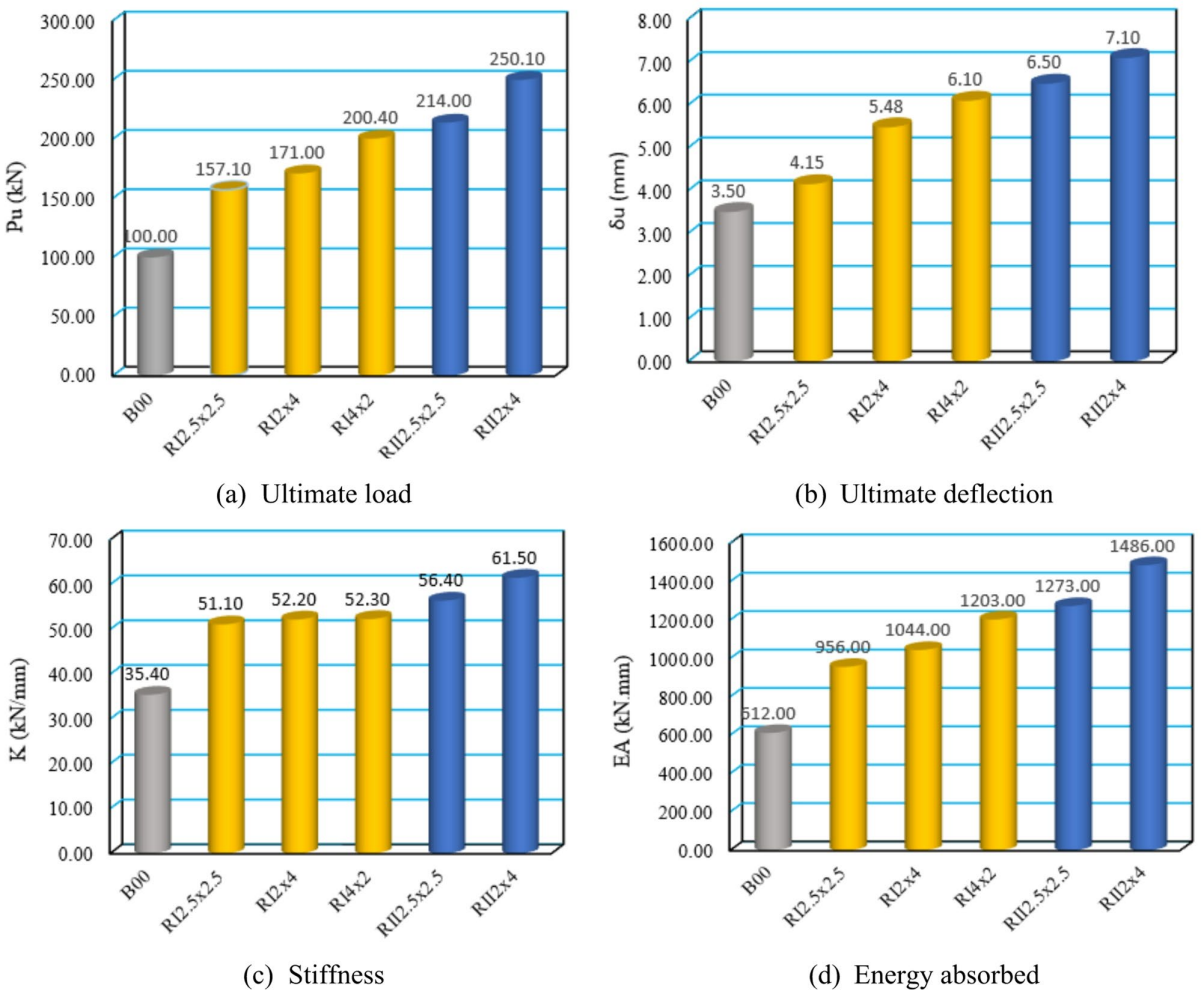
Effect of increasing rectangular tubes reinforcement ratio on performance of tested beams.

Group GII investigates the influence of increasing the steel tube-to-beam section ratio (ρ) on the structural behavior of RC beams with internal voids, as illustrated in Fig. 9. The experimental results reveal a strong and consistent improvement in structural performance with increasing ρ, despite the simultaneous increase in void ratio. Compared with B00, RI2.5 × 2.5 exhibited marked gains in all response parameters, confirming that even a modest inclusion of steel tubes can effectively counteract the weakening effect of internal openings. More importantly, the observed improvement trend remained consistent even at higher reinforcement levels. This indicates that the enhancement is not limited to initial cracking behavior but also contributes to overall beam capacity. The most pronounced response was observed in RII2 × 4, which resulted in increases of 150.10% in $\:{P}_{u}$, 102.86% in $\:{\delta\:}_{u}$, 73.73% in stiffness, and 142.81% in energy absorption relative to B00. The improvement in deflection behavior is particularly significant. The consistent increase in δ_u_ with rising ρ indicates that the beams became progressively more ductile, rather than simply stronger. In the low-reinforcement case, failure is governed by rapid stiffness degradation after cracking, with limited post-peak deformation. As ρ increases, the embedded steel tubes begin to act as internal load-carrying paths that remain effective after cracking the surrounding concrete. This delays localization of strains and redistributes internal stresses, allowing the beam to sustain larger curvatures before failure. In this way, the increase in deflection capacity is directly linked to improved crack control and enhanced post-cracking stability, rather than a reduction in stiffness alone. From a stiffness perspective, the presence of steel tubes increases the effective flexural rigidity of the cracked section, especially in the early loading stages, by reducing stress concentration around void edges. However, unlike conventional stiffening, this increase in stiffness does not lead to brittle behavior. Instead, the system maintains a balance between rigidity and deformability, which explains the simultaneous increase in both k and δ_u_ observed experimentally. Mechanistically, these enhancements are governed by stress redistribution around voids, confinement provided by the steel tubes, and crack-bridging action along potential failure planes. The interaction between concrete and steel becomes more pronounced as ρ increases, resulting in a composite-like behavior where the steel tubes partially substitute for the lost concrete in resisting both flexural tension and compression struts.

#### Effect of tube orientation (group GIII)

Group GIII investigates the influence of tube orientation on the structural performance of reinforced concrete beams with internal rectangular voids. The results clearly demonstrate that tube orientation has a significant effect on structural response, particularly in flexural behavior and deflection characteristics. Despite identical β and ρ values, RI4 × 2 exhibited superior performance, with a 17.19% increase in P_u_, an 11.31% increase in δ_u_, and a 15.23% increase in energy absorption, while stiffness showed only a negligible change (0.19%). The enhanced performance of RI4 × 2 can be explained by its more efficient horizontal tube configuration, which improves the distribution of flexural stresses and increases the effective moment of inertia about the principal bending axis. This configuration delays crack initiation and propagation in the tension zone, resulting in a more stable load–deflection response and improved deformation capacity. In contrast, RI2 × 4, with a less favorable orientation, experiences earlier localized stress concentrations, which limits its ability to sustain higher deformation levels. The nearly unchanged stiffness indicates that initial elastic behavior is governed mainly by the global section properties, while post-cracking behavior is strongly influenced by tube orientation.

#### Effect of increasing circular tubes reinforcement ratio (group GIV)

**Fig. 10:**
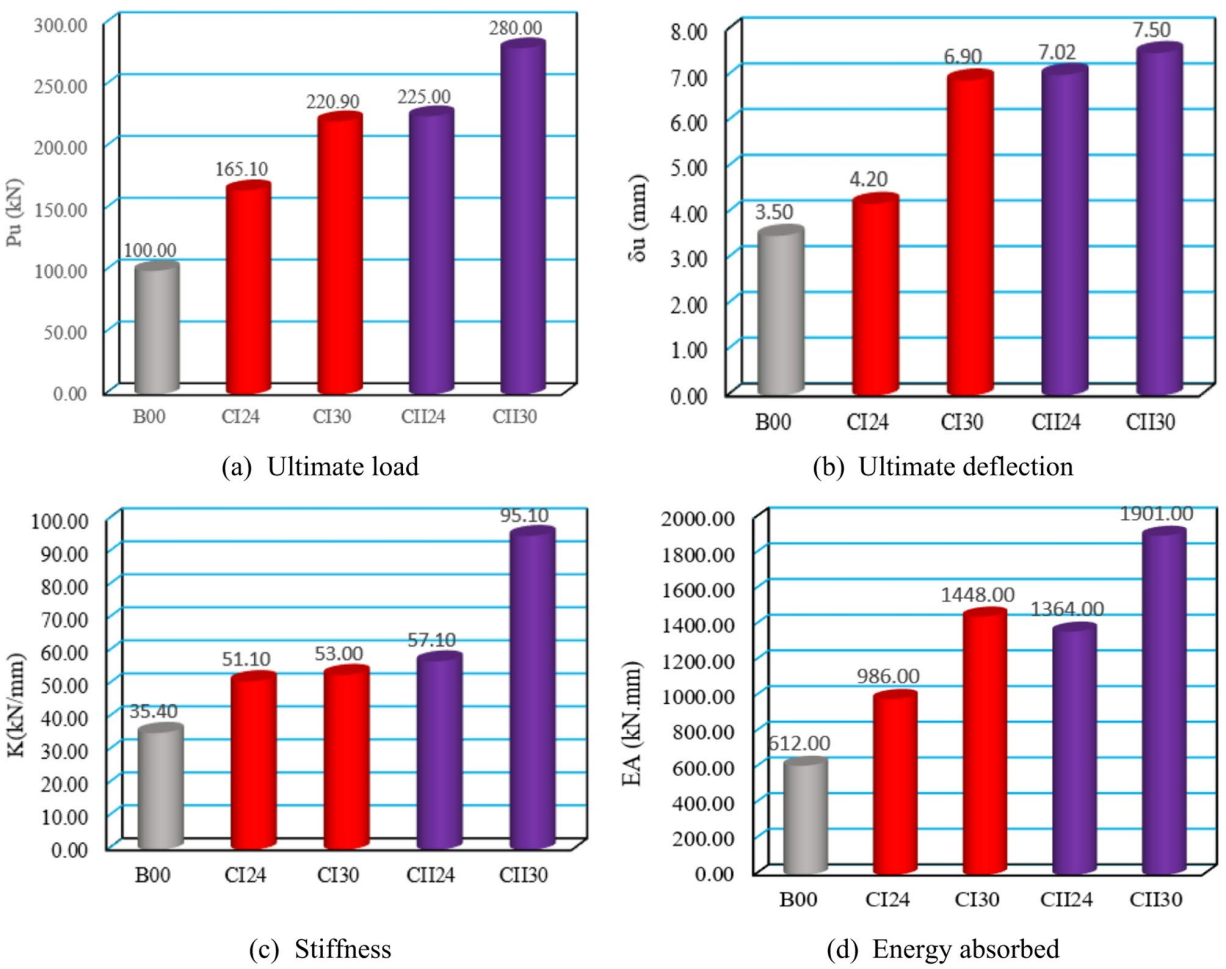
Effect of increasing circular tubes reinforcement ratio on performance of tested beams.

Group GIV examines the effect of increasing the circular steel tube reinforcement ratio (ρ) on the structural behavior of RC beams containing internal circular voids, as illustrated in Fig. 10. The experimental results demonstrate a substantial and nonlinear enhancement in structural performance with increasing circular tube reinforcement. Compared with the control specimen B00, the inclusion of circular steel tubes significantly improved load-carrying capacity, deformation response, stiffness, and energy absorption. The P_u_ capacity increased by 65.10% in CI24 and reached a maximum increase of 180.00% in CII30. Similarly, the δ_u_ increased progressively from 20.00% in CI24 to 114.29% in CII30, indicating a major enhancement in ductility and post-cracking deformation capacity. Unlike conventional brittle behavior observed in voided beams without reinforcement, the tube-reinforced specimens exhibited a more stable load–deflection response with delayed stiffness degradation after cracking. The improvement in deflection behavior is particularly important. As the reinforcement ratio increased, the beams were able to sustain larger deformations prior to failure while maintaining structural integrity. This behavior indicates that the embedded circular tubes effectively delayed crack localization and prevented sudden collapse mechanisms. The tubes continued to participate in load transfer after concrete cracking, thereby improving post-yield resistance and allowing gradual redistribution of internal stresses. Consequently, the increase in δ_u_ reflects enhanced ductility rather than simple flexibility, which is further supported by the simultaneous increase in stiffness. The stiffness (k) increased consistently with increasing ρ, reaching a maximum improvement of 168.64% in CII30. This enhancement is attributed to the higher flexural rigidity provided by the circular tubes, which compensate for the reduction in concrete area caused by the voids. In addition, the smooth circular geometry promotes more uniform stress flow around the openings, reducing stress concentrations that commonly initiate premature cracking in sharp-cornered voids. This stress redistribution mechanism contributes to improved crack control and more efficient utilization of the surrounding concrete section. Energy absorption (EA) also increased remarkably, with CII30 exhibiting a 210.62% increase compared with B00. This substantial gain confirms the effectiveness of circular steel tubes in improving structural toughness and deformation sustainability. The enhanced energy dissipation results from the combined action of crack bridging, confinement, and the ability of the steel tubes to maintain load resistance during advanced stages of deformation.

It is noted that the circular tube reinforcement ratio (ρ) was increased by simultaneously increasing both the tube diameter and wall thickness (from 1.7 mm for CI24 and CII24 to 2.4 mm for CI30 and CII30, as shown in Table 3). Therefore, the observed improvements in P_u_, δ_u_, k, and EA for circular tube specimens reflect the combined contribution of increased steel area, larger diameter, and greater wall thickness.

#### Effect of tube type (group GV)

Group GV evaluates the effect of tube geometry—rectangular versus circular—on the structural performance of RC beams containing internal voids. The experimental results revealed that CI24 achieved slightly higher structural performance despite having a lower reinforcement ratio. Compared with RI2.5 × 2.5, the CI24 beam exhibited increases of 5.09% in ultimate load capacity and 3.14% in energy absorption, while stiffness and ultimate deflection remained nearly unchanged. The similarity in stiffness and δ_u_ indicates that both tube types provided comparable resistance to deformation and maintained stable load–deflection behavior throughout loading. However, the slightly higher load capacity and toughness of the circular tube specimen suggest that tube geometry influences the efficiency of stress transfer within the beam. The improved behavior of the circular tube can be attributed primarily to its smoother geometric profile, which promotes more uniform stress distribution around the void region and minimizes localized stress concentrations. Unlike rectangular tubes with sharp corners that tend to generate stress intensification zones, circular tubes facilitate continuous stress flow and more effective crack redistribution. This behavior delays crack propagation and contributes to improved post-cracking performance and energy dissipation. Regarding deflection behavior, both specimens exhibited similar ultimate deflections, indicating comparable ductility levels. Nevertheless, the circular tube specimen demonstrated a more stable deformation response during the later loading stages, which can be linked to the enhanced confinement and reduced stress concentration provided by the circular geometry. This contributed to improved deformation sustainability without excessive stiffness loss after cracking.

## FEM

Nonlinear three-dimensional finite element modeling (FEM) incorporating both geometric and material nonlinearities were performed using ABAQUS. ABAQUS is a widely recognized and powerful finite element software package that has been extensively applied in various engineering disciplines, including structural, civil, mechanical, aerospace, and geotechnical engineering^63–65^.

### Geometric configuration, load application method, and boundary conditions

**Fig. 11:**
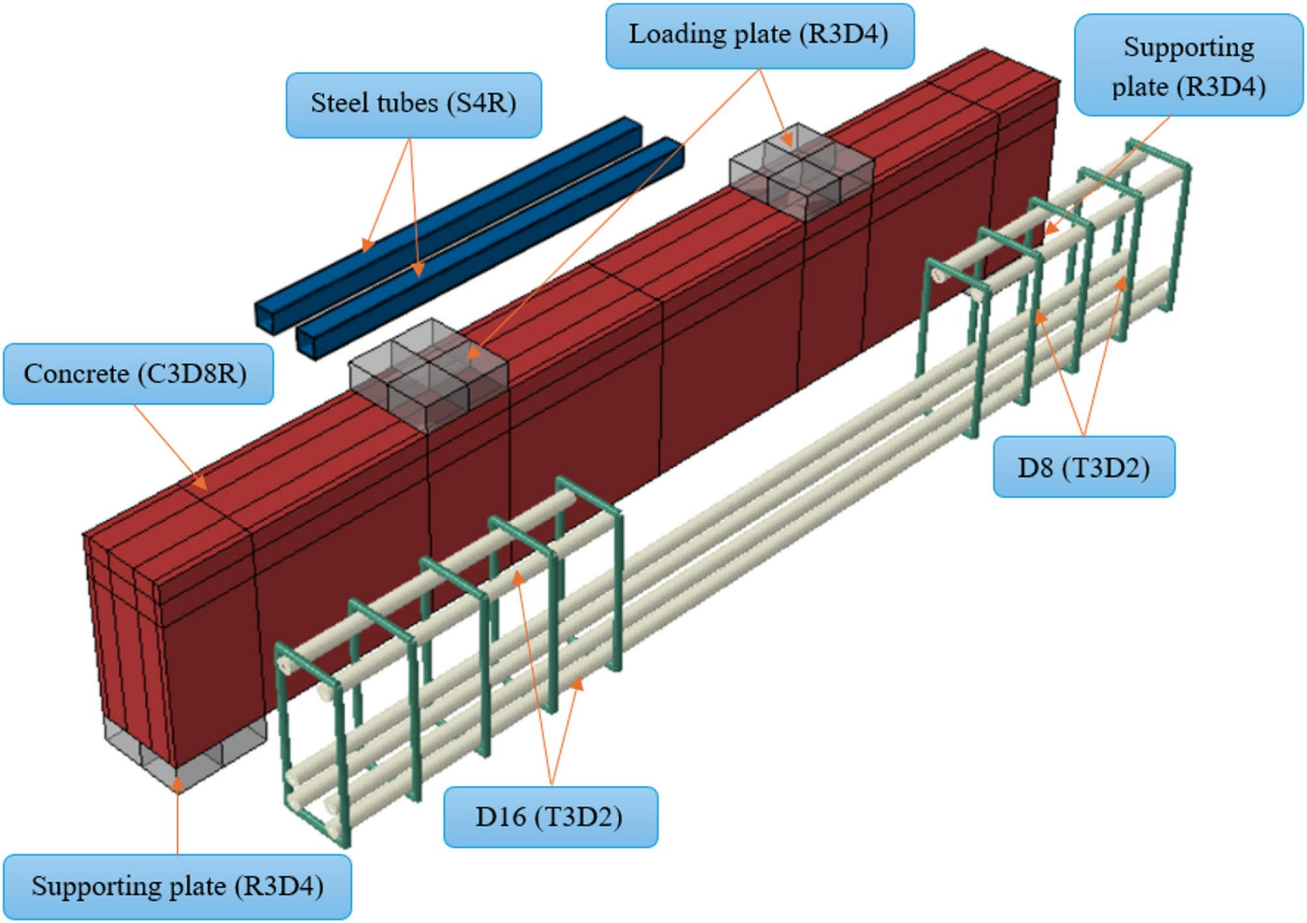
Element type of beam RII2.5 × 2.5.

**Fig. 12:**
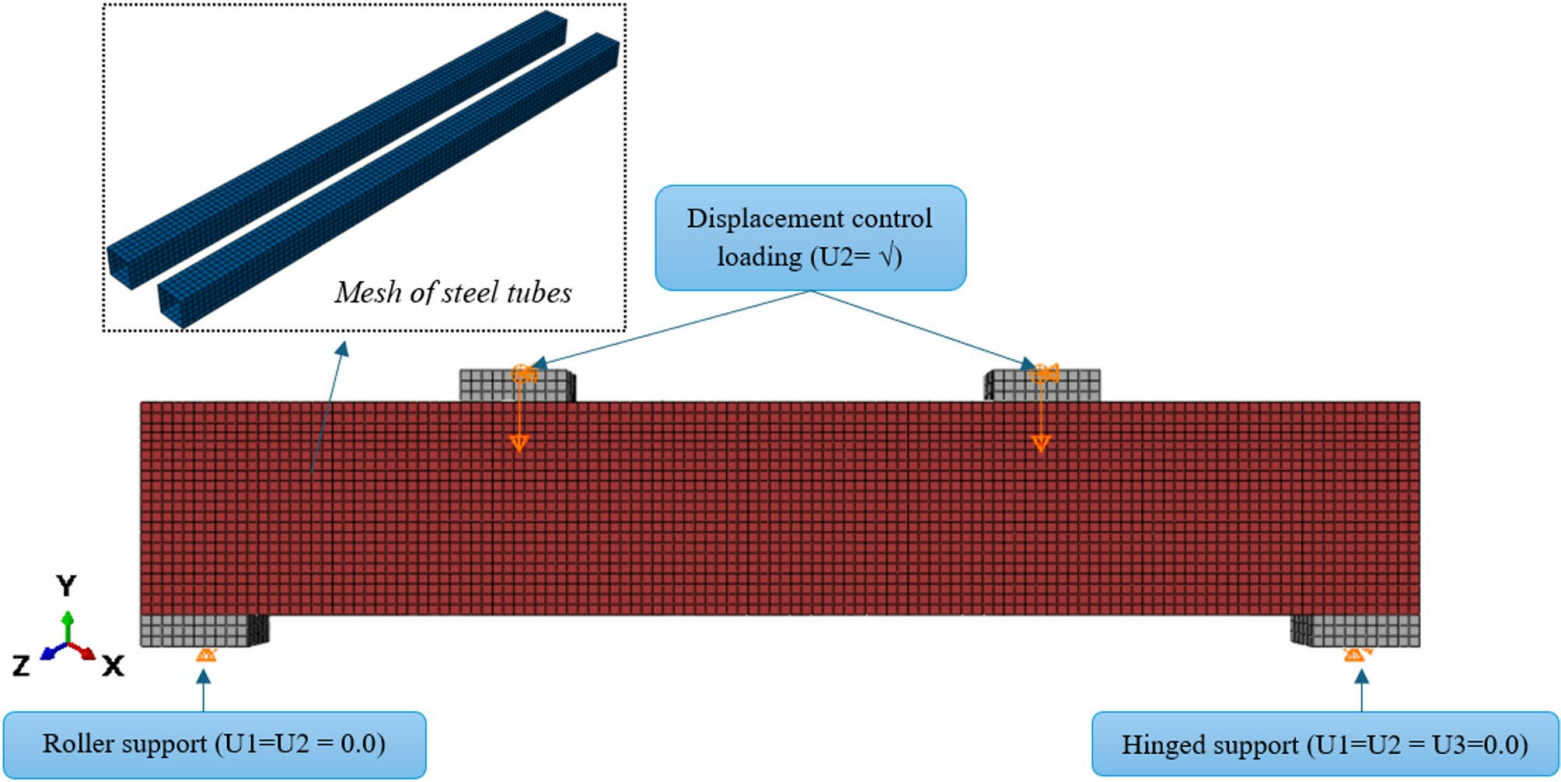
Mesh layout, boundary constraints, and loading setup for the tested beams.

**Fig. 13:**
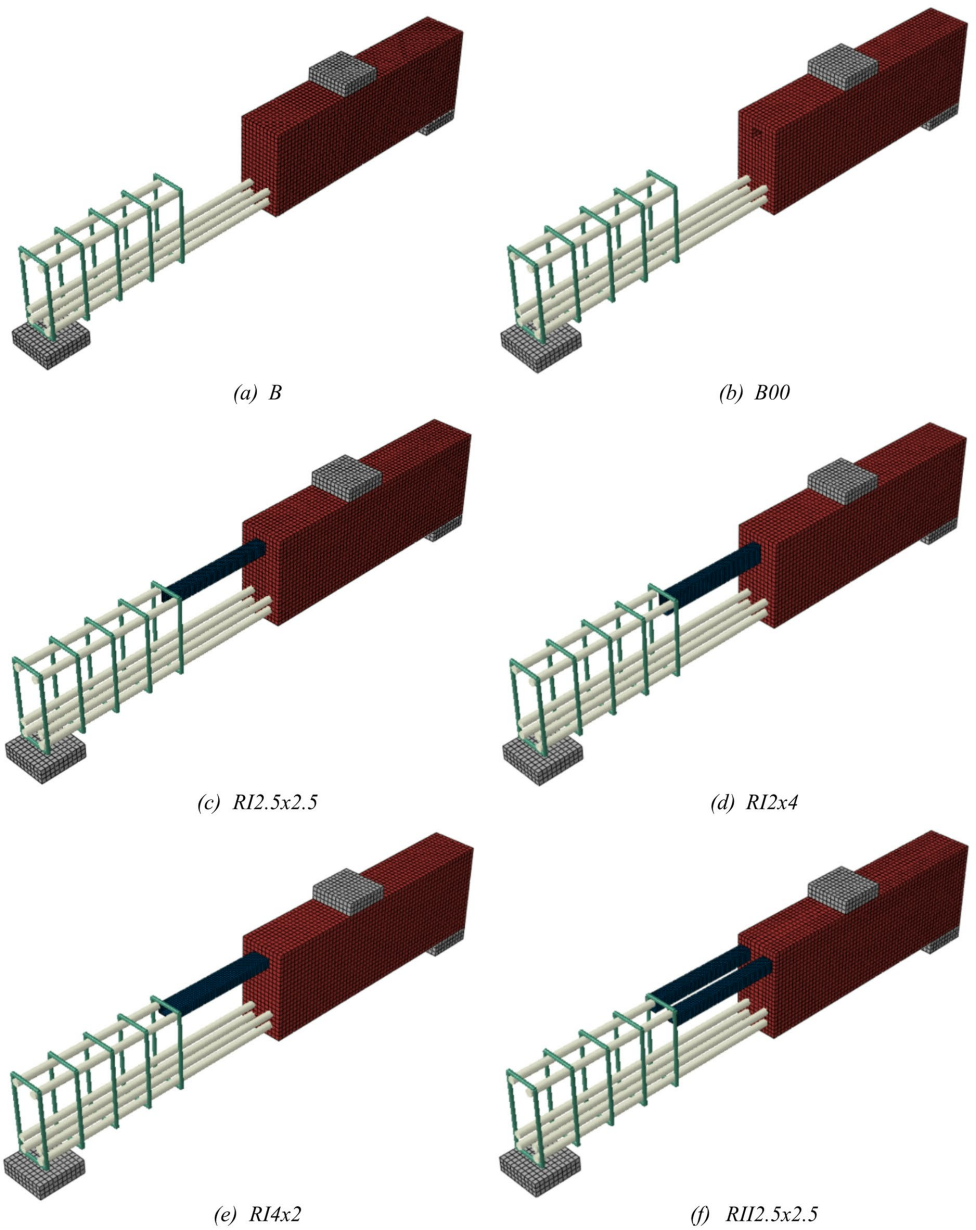

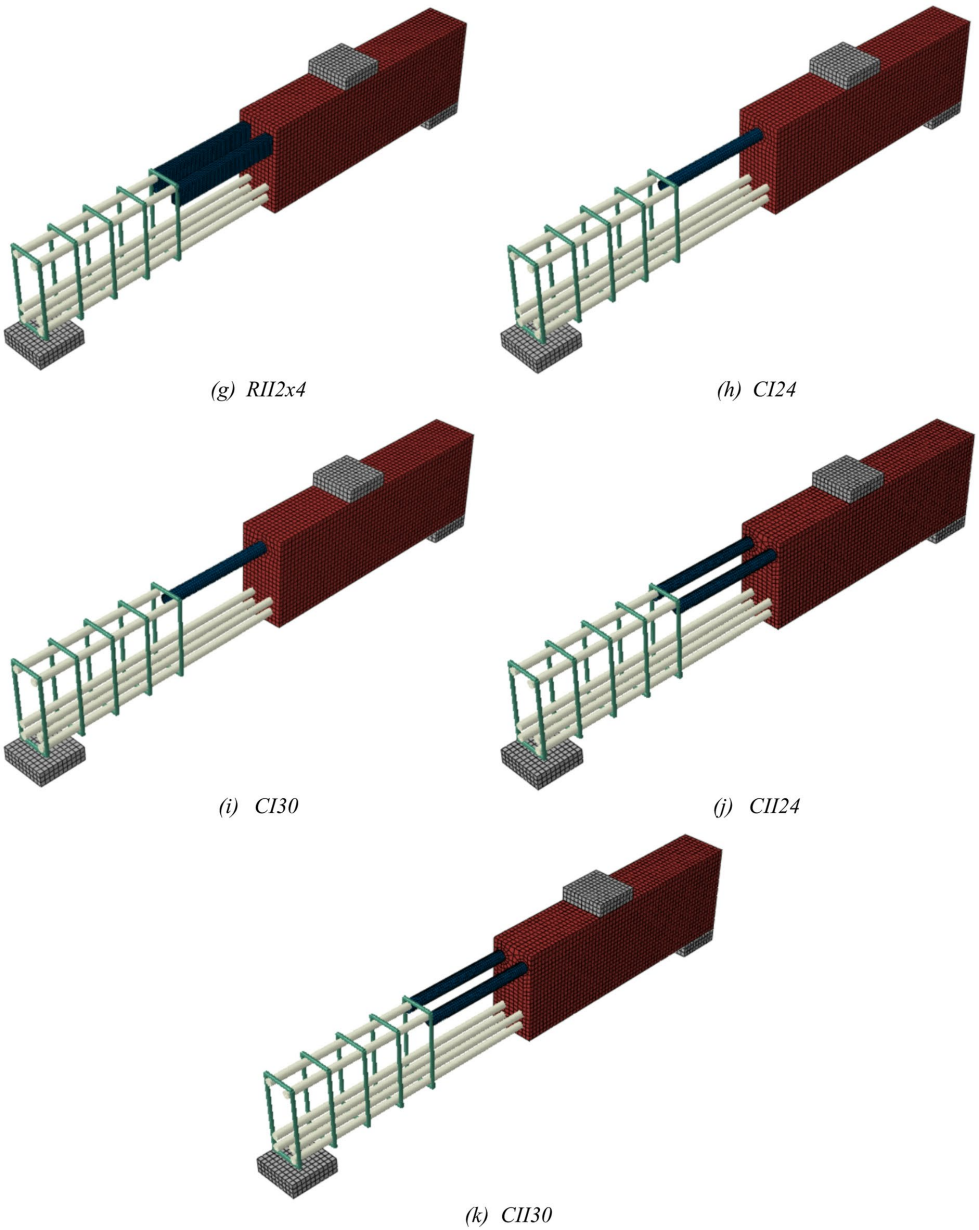
FE models of all tested beams.

Based on the experimental observations, a three-dimensional finite element model (FEM) of beam RII2.5 × 2.5 was developed, as illustrated in Fig. 11. The concrete was modeled using eight-node linear brick elements with reduced integration (C3D8R), while the loading and supporting plates were simulated using four-node rigid surface elements (R3D4). Longitudinal reinforcement and stirrups were represented by two-node truss elements (T3D2), whereas the embedded steel tubes were modeled using four-node shell elements (S4R) to capture their thin-walled behavior accurately. To replicate the experimental conditions, rigid steel plates were incorporated at the loading and support regions, maintaining the actual shear span geometry. Boundary conditions were defined along a central line beneath each support plate to simulate line contact, with hinged and roller constraints representing the support system. Loading was applied through two reference points at midspan, tied to the loading plates using multi-point constraints to ensure uniform load transfer and minimize stress concentrations (Fig. 12). The finite element models for all tested beams are presented in Fig. 13.

### Contact and Interaction definitions between elements

The FE model was developed with carefully defined interaction conditions to accurately replicate the experimental behavior. A tie constraint was applied between the concrete beam and both the loading and support plates to ensure rigid connectivity, effectively preventing any relative displacement or rotation at the interfaces. The longitudinal reinforcement and stirrups were modeled using the embedded element approach, which enforces full strain compatibility and displacement continuity with the surrounding concrete. In addition, a tie constraint was assigned between the embedded steel tubes and the surrounding concrete core to simulate a fully bonded interface condition. This assumption was considered reasonable because no noticeable interface slip or separation was observed during the experimental testing. Furthermore, the tie-constraint approach has been extensively adopted in previous finite element investigations of concrete–steel composite members to ensure full strain compatibility, displacement continuity, and enhanced numerical stability^66–71^.

### Sensitivity analysis

**Fig. 14:**
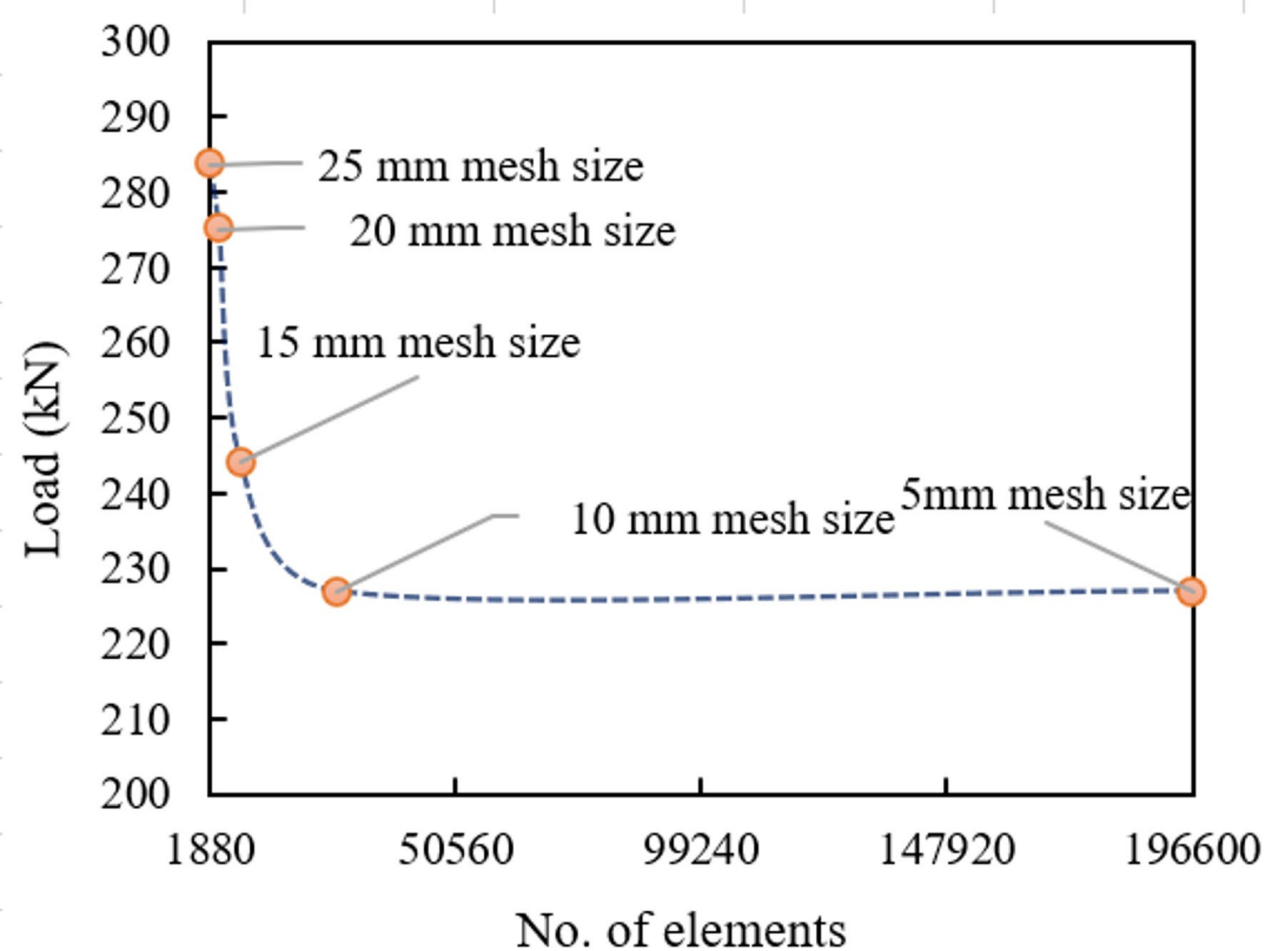
Load level for different mesh sizes of beam RII2.5 × 2.5.

Mesh density plays a crucial role in balancing numerical accuracy and computational efficiency in finite element analysis. In this study, mesh sizes of 5 mm to 25 mm were examined to evaluate mesh sensitivity. The coarsest mesh (25 mm) overestimated the load-carrying capacity of specimen RII2.5 × 2.5, while finer meshes (5–10 mm) produced similar peak load results. Among them, the 10 mm mesh showed the best agreement with the experimental post-peak response. Therefore, a 10 mm mesh was adopted for all subsequent analyses to ensure an optimal balance between accuracy and computational cost. (Fig. 14)

### Material constitutive modeling

#### Concrete

**Fig. 15:**
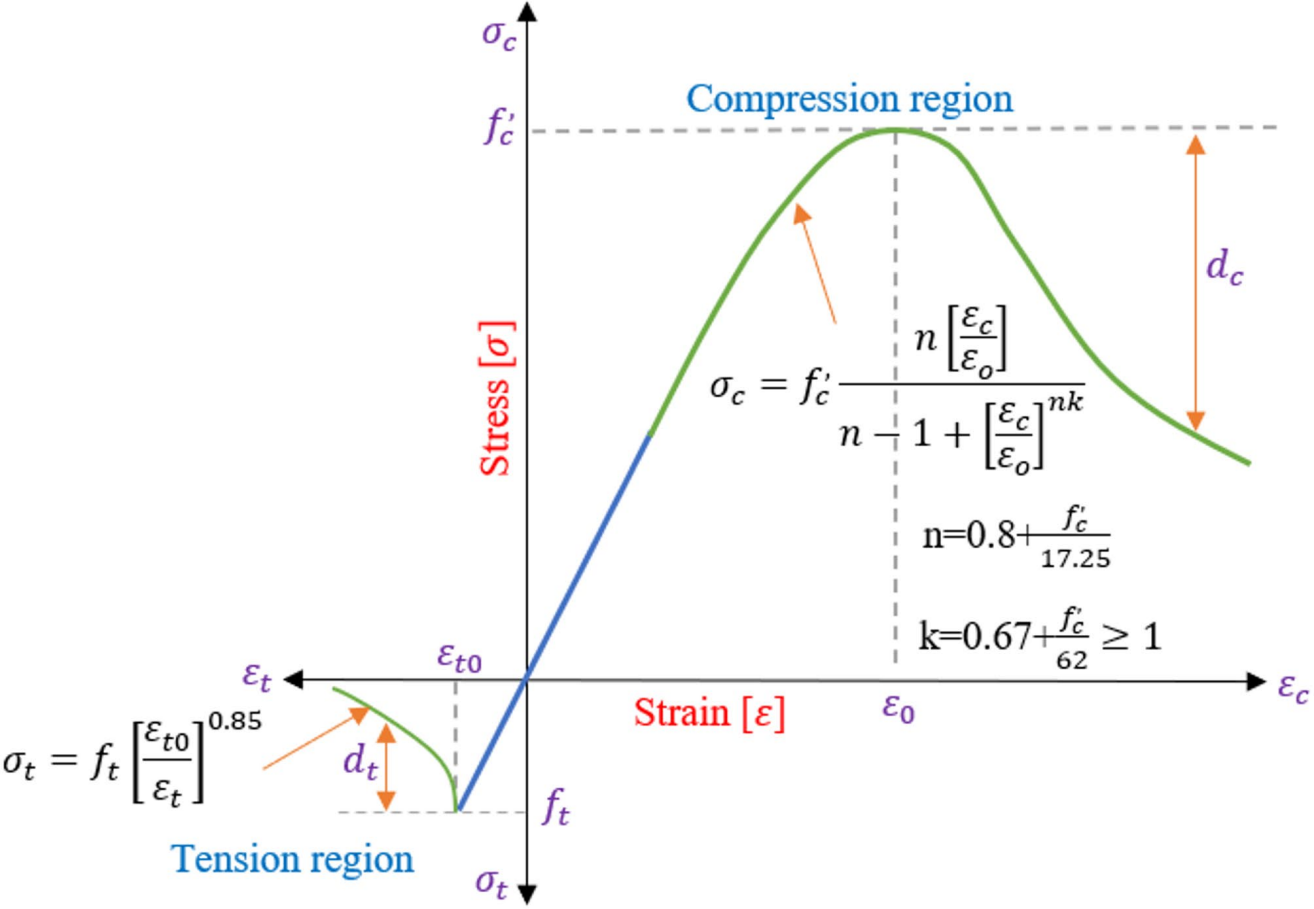
Mechanical behavior of concrete under both compressive and tensile loading^72^.

The nonlinear and inelastic behavior of concrete was simulated using the concrete damaged plasticity (CDP) model in ABAQUS, selected over alternative approaches due to its proven accuracy and numerical stability. The CDP model requires detailed input parameters, including uniaxial stress–strain relationships in tension and compression, damage parameters ($\:{d}_{t}$ and $\:{d}_{c}$), and plasticity constants. These values were adopted from^72^, with the corresponding stress–strain curve presented in Fig. 15. This modeling approach enabled a realistic representation of concrete response under complex loading scenarios within the finite element framework. To define the compressive behavior of concrete in ABAQUS, the inelastic strain $\:{\varepsilon\:}_{c}^{\sim in}$ is first specified along with the associated stress $\:{\sigma\:}_{c}$, as described in Eq. (1). The software then calculates the corresponding plastic strain $\:{\varepsilon\:}_{c}^{\sim pl}$ by incorporating the damage effects induced by compressive loading, following Eq. (2).

1 $$\:{\varepsilon\:}_{c}^{\sim in}={\varepsilon\:}_{c}-{\varepsilon\:}_{oc}^{\sim pl}={\varepsilon\:}_{c}-\frac{{\sigma\:}_{c}}{{E}_{0}}$$

2 $$\:{\varepsilon\:}_{c}^{\sim pl}={\varepsilon\:}_{c}^{\sim in}-\frac{{d}_{c}}{(1-{d}_{c})}$$

The tensile stress–strain response is defined by specifying the tensile stress $\:{\sigma\:}_{t}$ and the inelastic cracking strain $\:{\varepsilon\:}_{t}^{\sim ck}$. These parameters are then used to compute the corresponding plastic tensile strain $\:{\varepsilon\:}_{t}^{\sim pl}$ using Eq. (3), which accounts for the damage variable $\:{d}_{t}$ and the elastic modulus E_0_:

3 $$\:{\varepsilon\:}_{t}^{\sim pl}={\varepsilon\:}_{t}^{\sim ck}-\frac{{d}_{t}}{(1-{d}_{t})}\frac{{\sigma\:}_{t}}{{E}_{o}}$$

In the Concrete Damaged Plasticity model, four essential parameters were defined: dilation angle, eccentricity (ε), shape factor $\:{K}_{c}$, and the compressive strength ratio ($\:{f}_{bo}/{f}_{co}$). The dilation angle influences the failure surface’s inclination, while ε governs its rate of curvature. The $\:{K}_{c}$ parameter modifies the deviatoric section’s geometry, and the strength ratio represents concrete performance under biaxial versus uniaxial loading. For the purposes of this analysis, values of 26, 0.1, 0.67, and 1.16 were used as per^72^. To improve convergence and address mesh sensitivity, a viscosity parameter of 0.0001 was also incorporated.

#### Reinforcement materials

The reinforcement was modeled using a bilinear elastic–plastic constitutive law with isotropic hardening to capture nonlinear behavior under loadings^73,74^. The stress–strain relationship followed Han and Huo’s two-segment model^75^, with elastic properties derived from experimental data. A 1% hardening modulus was adopted to represent post-yield behavior, ensuring accurate simulation of material response under monotonic loading conditions based on the formulation in^76^.

4 $$\:{\sigma\:}_{i}={E}_{s}{\varepsilon\:}_{s}\:\:\:\:\:\:\:\:\:\:\:\:\:\:\:\:\:\:\:\:\:\:\:\:\:\:\:\:\:\:\:\:\:\:for\:{\varepsilon\:}_{s}\le\:{\varepsilon\:}_{sy}\:\:\:\:\:\:\:\:\:\:\:\:\:\:\:\left(elastic\:region\right)\:$$

5 $$\:{\sigma\:}_{i}={f}_{sy}+{E}_{p}({\varepsilon\:}_{s}-{\varepsilon\:}_{sy})\:\:\:\:\:\:\:for\:{\varepsilon\:}_{s}\le\:{\varepsilon\:}_{sy}\:\:\:\:\:\:\:\:\:\:\:\:\:\:\:\left(plastic\:region\right)$$

In ABAQUS, steel was modeled as linearly elastic until the yield point and plastic beyond it. The required parameters—$\:{\sigma\:}_{i}$, $\:{E}_{s}$, $\:{\varepsilon\:}_{s}$, $\:{f}_{sy}$, and $\:{\varepsilon\:}_{sy}$ —were used to define the material law. Engineering stress-strain data were converted to true values using equations from^77^:

6 $$\:{f}_{tr}={f}_{norm}(1+{\varepsilon\:}_{norm})$$

7 $$\:{\varepsilon\:}_{tr}^{pl}=ln\left(1+{\varepsilon\:}_{norm}\right)-{f}_{tr}/{E}_{s}$$

In this context, $\:{f}_{tr}$ refers to the true stress and $\:{f}_{norm}$ to the nominal (engineering) stress. Similarly, $\:{\varepsilon\:}_{tr}^{pl}$ indicates the true plastic strain, and $\:{\varepsilon\:}_{norm}$ represents the nominal strain.

### Verifications

**Fig. 16:**
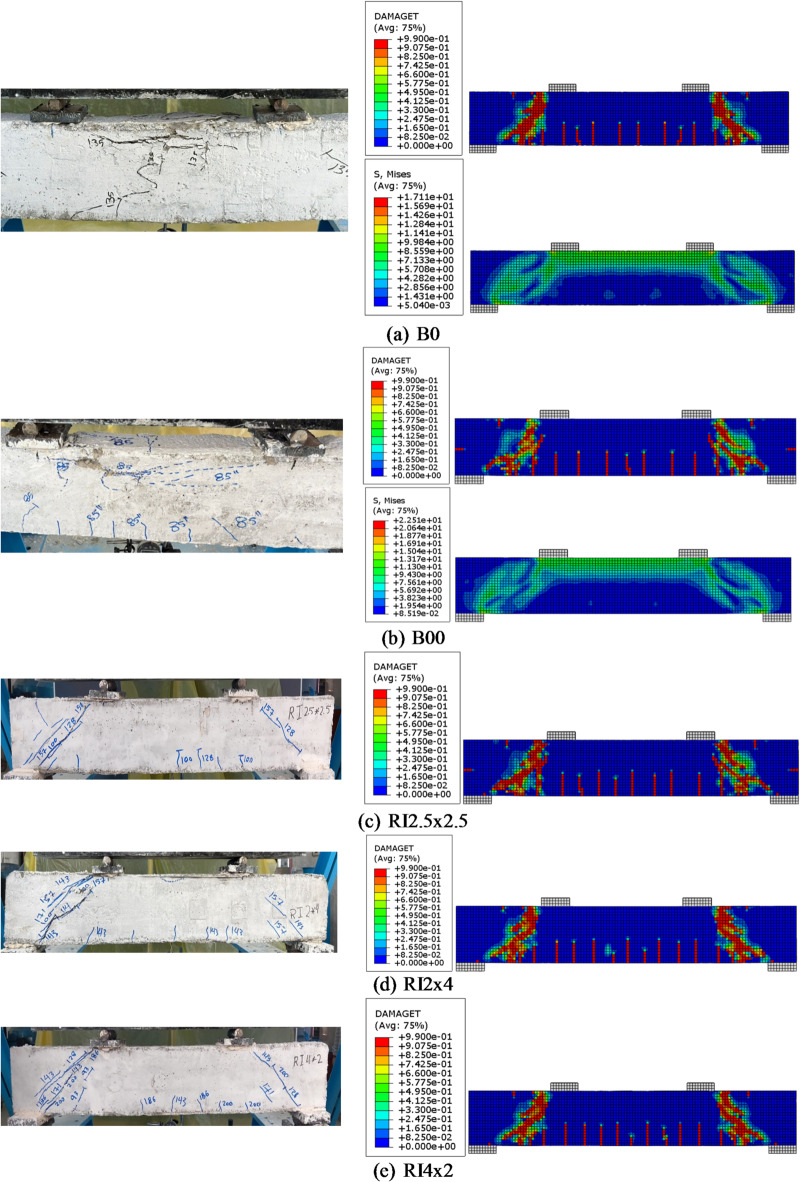

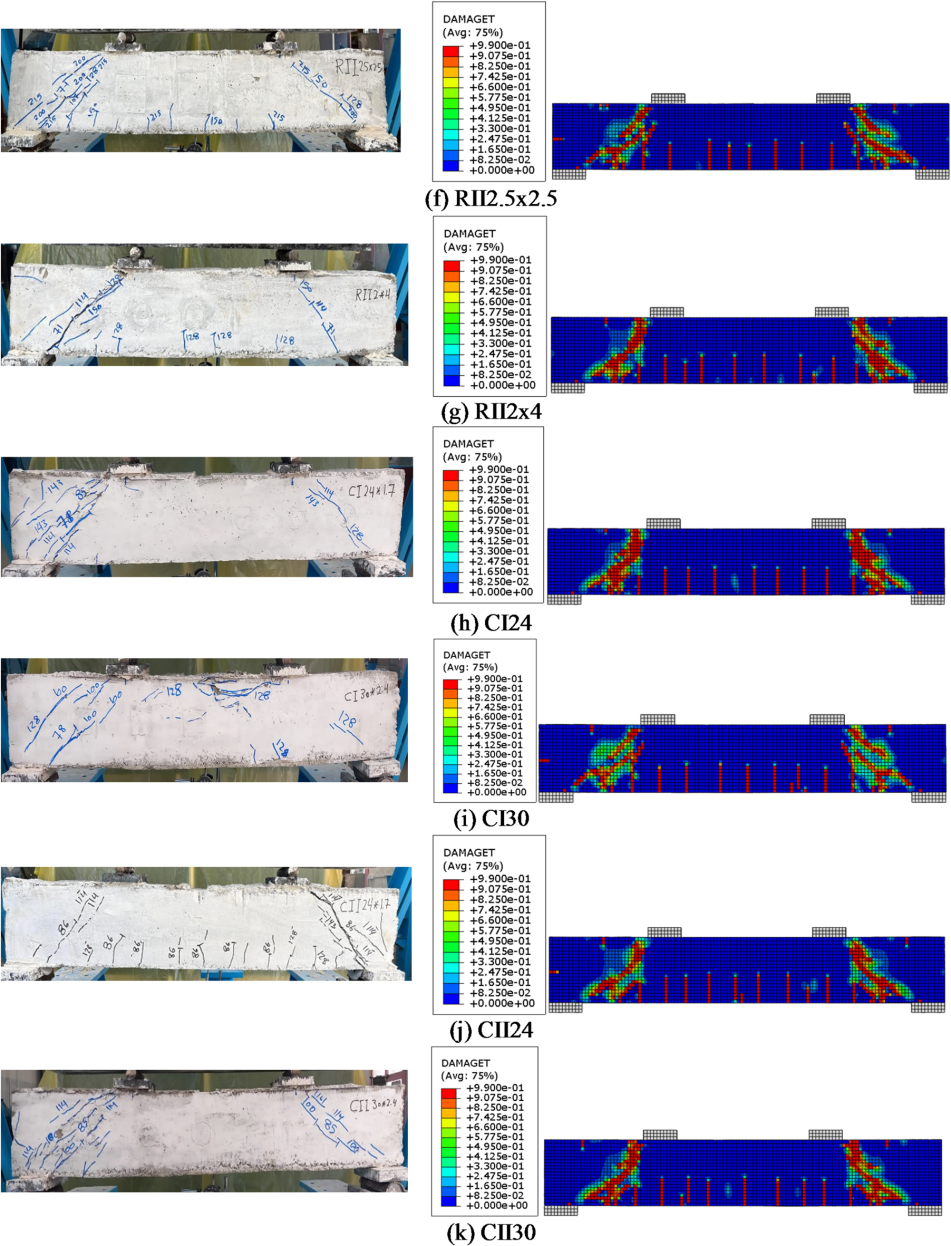
Experimental vs. Numerical failure patterns based on tensile damage (DAMAGET).

**Fig. 17:**
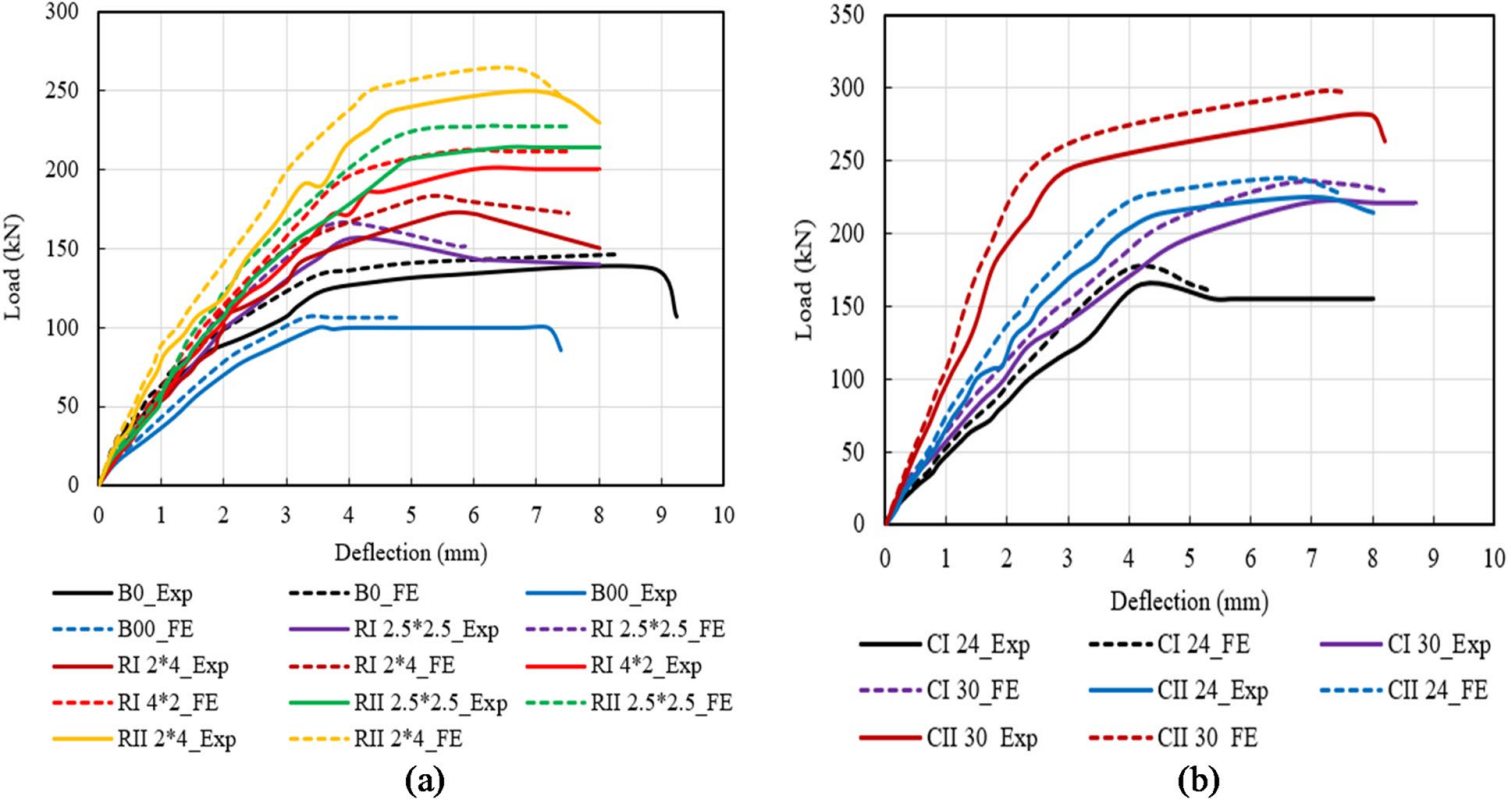
Load–deflection curves: experimental results vs. numerical predictions.

The finite element results were systematically compared with experimental findings for all test specimens. As illustrated in Figs. 16 and 17, the model accurately replicates the failure patterns and load–deflection response. The numerical simulations captured compression-flexure and shear failure modes, including the formation of inclined shear cracks with orientations closely matching those observed experimentally. Crack locations were also well predicted. As presented in Table 5, the average ratios of experimental to finite element ($\:Exp/FE$) values (µ) were 0.954 for load and 1.051 for deflection at failure. The corresponding standard deviations were 0.014 and 0.092, while the coefficients of variation (CoV) were 0.015 and 0.088, respectively. These results confirm that the FE model offers high accuracy in simulating both global and local structural responses. The modeling approach demonstrates robustness and is well-suited for extension to other structural configurations or loading conditions.

**Table 5:** Validation of numerical predictions against experimental data.

Beam’s ID	$\:{P}_{U}\:\left(kN\right)$			$\:{\delta\:}_{u}\:\left(mm\right)$		
	$\:Exp$	$\:FE$	$\:Exp/FE$	$\:Exp$	$\:FE$	$\:Exp/FE$
B0	138.0	140.0	0.986	4.30	4.90	0.878
B00	100.0	105.0	0.952	3.50	3.20	1.094
RI2.5 × 2.5	157.10	166.0	0.946	4.15	3.70	1.122
RI2 × 4	171.0	180.0	0.950	5.48	5.80	0.945
RI4 × 2	200.40	212.0	0.945	6.10	6.70	0.910
RII2.5 × 2.5	214.0	227.0	0.943	6.50	6.00	1.083
RII2 × 4	250.10	265.0	0.944	7.10	6.40	1.109
CI24	165.10	174.0	0.949	4.20	3.70	1.135
CI30	220.90	232.0	0.952	6.90	6.30	1.095
CII24	225.0	230.0	0.978	7.02	6.43	1.092
CII30	280.0	294.0	0.952	7.50	6.85	1.095
µ			0.954			1.051
SD			0.014			0.092
CoV			0.015			0.088

µ: Average; SD: Standard Deviation; CoV: Coefficient of Variation.

## Parametric study

A parametric study was conducted to evaluated the influence of concrete compressive strength on ultimate load capacity. The analysis included beam B00 as a control beam, which contained a single square void (25 × 25 mm), representing a β ratio of 3.13%, but with no embedded tube. Four different values of $\:{f}_{c}$ —20 MPa, 25 MPa, 35 MPa, and 45 MPa—were evaluated.

**Fig. 18:**
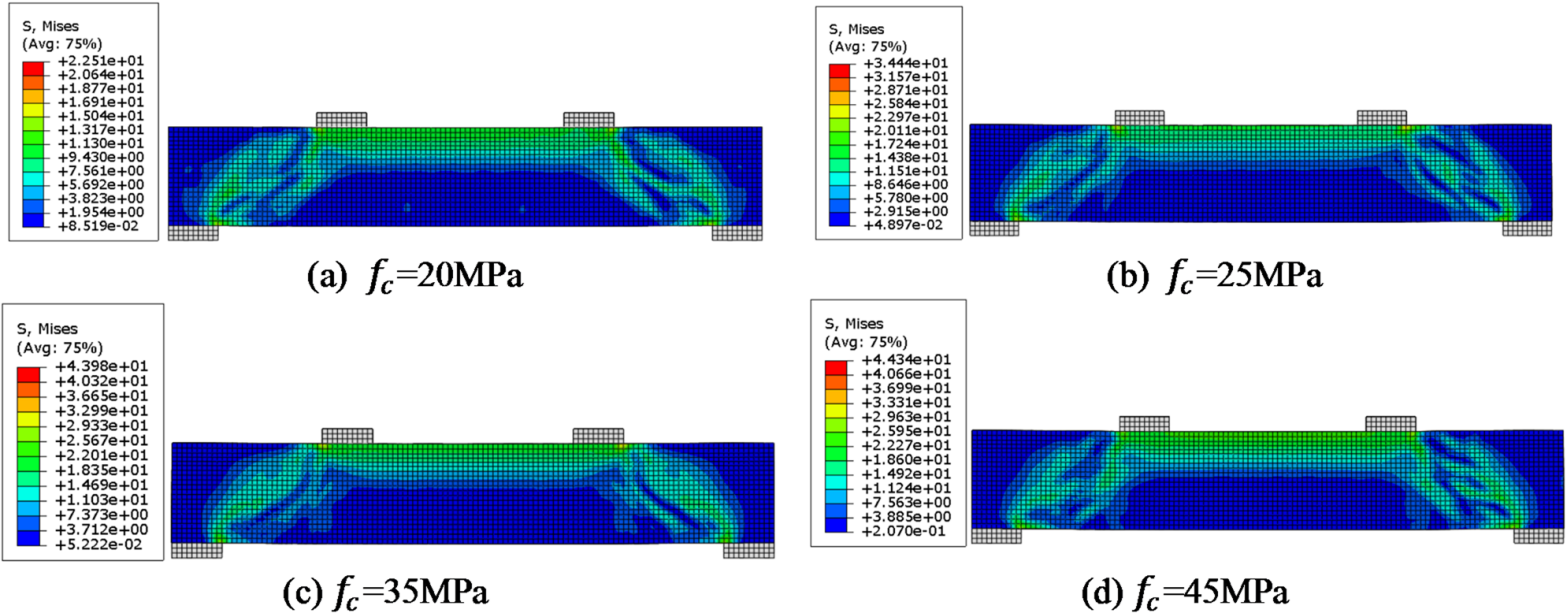
Influence of concrete compressive strength on failure modes based on von Mises stress.

**Fig. 19:**
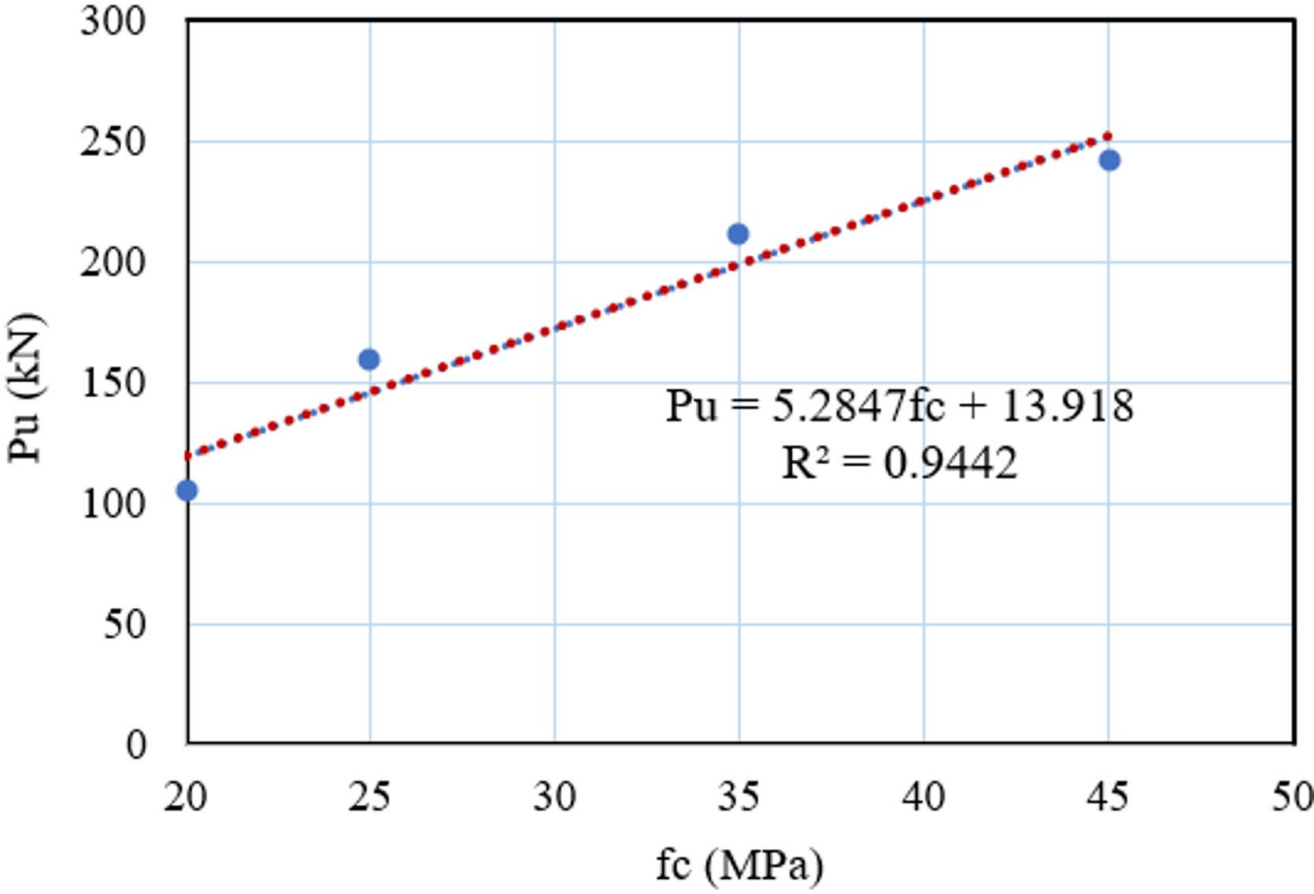
Influence of concrete compressive strength ($\:{f}_{c}$) on the ultimate load capacity.

The results demonstrate that $\:{f}_{c}$ has a significant impact on the structural performance of RC beams, particularly in terms of failure modes, which are consistent with the experimental observations (Fig. 18). Furthermore, $\:{f}_{c}$ strongly influences the ultimate load capacity ($\:{P}_{u}$) of the tested beams, as shown in Fig. 19, with a clear upward trend observed as $\:{f}_{c}$ increases. Specifically, $\:{P}_{u}$ increases by approximately 230% when $\:{f}_{c}$ rises from 20 MPa to 45 MPa. The relationship between $\:{P}_{u}$ and $\:{f}_{c}$ can be expressed by the following equation:

8 $$\:{P}_{u}=5.28{f}_{c}+13.92$$

## Conclusion

This study presented an experimental and numerical investigation on eleven RC beams with longitudinal voids strengthened using embedded steel tubes (rectangular and circular sections), tested under four-point bending. The specimens included a solid control beam, a void-only beam, and nine strengthened configurations with varying void ratios (β = 2.26%–8.00%) and reinforcement ratios (ρ = 0%–16.96%). Based on the results, the following conclusions are drawn:

1. The introduction of longitudinal voids in the compression zone significantly degrades structural performance. Compared with the solid beam, the void-only specimen exhibited a 27.5% reduction in ultimate load (P_u_), while stiffness (k) and energy absorption (EA) decreased by approximately 41% and 41%, respectively, confirming reduced flexural efficiency and increased stress concentration effects.
2. Embedding steel tubes within voids effectively restores and enhances structural performance. Across all tested specimens, improvements ranged from 57.1% to 180.0% in P_u_, 18.6% to 114.3% in δ_u_, 44.3% to 168.6% in k, and 56.2% to 210.6% in EA, compared with the unreinforced void beam. These enhancements demonstrate substantial gains in both strength and ductility.
3. Rectangular tube reinforcement showed a strong positive correlation between reinforcement ratio and performance. The highest-performing configuration (RII2 × 4, ρ = 12.8%, β = 8.0%) achieved P_u_ = 250.1 kN, representing + 150.1% over the void beam and + 81% over the solid beam, along with + 102.9% in δ_u_ and + 142.8% in EA. This specimen showed the greatest improvements in load capacity, stiffness, and overall structural efficiency.
4. Circular tube configurations provided the highest overall performance. The CII30 specimen (ρ = 16.96%, β = 7.07%) reached P_u_ = 280.0 kN, corresponding to + 180% over the void beam and + 103% over the solid beam, with + 114.3% increase in δ_u_ and + 210.6% increase in EA. It also exhibited the highest energy absorption capacity, confirming superior structural toughness and dissipation performance.
5. A clear geometric effect was observed in rectangular tubes: horizontally oriented tubes improved performance by 17.2% in P_u_ compared to vertical orientation, due to a higher effective moment of inertia and improved stress distribution.
6. Circular tubes exhibited approximately 5% higher P_u_ and EA compared to rectangular tubes of similar size, attributed to uniform stress flow and reduced stress concentration around void boundaries.
7. The validated 3D nonlinear FE model showed excellent agreement with experimental results, with average ratios of Exp/FE = 0.954 for P_u_ and 1.051 for δ_u_, confirming high predictive accuracy in both strength and deformation response.

The findings provide a viable structural solution for incorporating service voids within RC beams while maintaining or enhancing structural capacity. Embedded steel tubes can be adopted in modern building systems to integrate electrical and mechanical services without compromising beam depth efficiency. The proposed strengthening technique is applicable in high-rise buildings, bridges, and retrofitting of existing structures where service integration, space optimization, and structural enhancement are required.

### Limitation and future recommendations

Despite the promising findings obtained in this study, several limitations should be acknowledged. First, the experimental program included only eleven beam specimens, and each beam was tested once without replicate specimens. Consequently, the variability and statistical scatter of the experimental results could not be quantified. Therefore, future studies are recommended to include replicate tests and statistical analyses to improve the reliability and repeatability of the conclusions. Second, the experimental measurements were primarily limited to mid-span deflection using a single dial gauge. Additional instrumentation, such as strain gauges for tensile reinforcement, concrete compression zones, and embedded steel tubes, was not included. As a result, direct verification of reinforcement yielding, concrete crushing behavior, and the degree of composite interaction between the steel tubes and surrounding concrete could not be fully quantified. Moreover, future investigations should employ more comprehensive instrumentation systems to provide a deeper understanding of the structural response, strain distribution, and crack propagation behavior of such composite beams. Finally, the experimental investigation was conducted using relatively small-scale beam specimens with dimensions of 100 × 200 mm and a span length of 1200 mm. In such specimens, the stiffness contribution of the embedded steel tubes may be proportionally greater than that expected in full-scale structural members. Therefore, future research should extend the investigation to larger beam dimensions and full-scale specimens, supported by advanced numerical simulations and parametric studies, to evaluate potential size effects and verify the applicability of the proposed strengthening system under real structural conditions.

## Data Availability

The raw data required to reproduce these findings are available only with direct contact through email to the corresponding author. The processed data required to reproduce these findings are available only with direct contact through email to the corresponding author.

## List of symbols

A_o_: Opening cross-sectional area (mm^2^)
A_t_: Steel tube cross-sectional area (mm^2^)
b: Beam width (mm)
h: Beam height (mm)
t: Tube wall thickness (mm)
N: Number of openings/tubes
p: Applied load (kN)
P_u_: Ultimate load capacity (kN)
δ: Deflection (mm)
δ_u_: Ultimate deflection (mm)
k: Elastic stiffness (kN/mm)
EA: Energy absorption (kN·mm)
β: Openings-to-beam section ratio (%)
ρ: Tubes-to-beam section ratio (%)
µ: Mean (average) value
SD: Standard deviation
CoV: Coefficient of variation
$\:{\sigma\:}_{c}$: Concrete compressive stress (MPa)
$\:{\sigma\:}_{i}$: Steel stress (MPa)
$\:{\sigma\:}_{t}$: Concrete tensile stress (MPa)
f_bo_/f_co_: Compressive strength ratio
f_c_: Concrete compressive strength (MPa)
f_co_: Uniaxial compressive strength of concrete (MPa)
f_bo_: Biaxial compressive strength of concrete (MPa)
f_norm_: Nominal (engineering) stress (MPa)
f_sy_: Steel yield stress (MPa)
f_tr_: True stress (MPa)
E_s_: Steel elastic modulus (GPa)
E_p_: Plastic hardening modulus of steel (GPa)
E_0_: Concrete elastic modulus (GPa)
d_c_: Compressive damage parameter
d_t_: Tensile damage parameter
K_c_: Shape factor of the deviatoric section (CDP model)
ε: Eccentricity parameter (CDP model)
ε_s_: Steel strain
ε_sy_: Steel yield strain
${\varepsilon}_{c}$: Concrete compressive strain
${\varepsilon}_{c}^{\sim in}$: Concrete inelastic compressive strain
${\varepsilon}_{c}^{\sim pl}$: Concrete plastic compressive strain
${\varepsilon}_{t}^{\sim ck}$: Concrete cracking strain in tension
${\varepsilon}_{t}^{\sim pl}$: Concrete plastic tensile strain
${\varepsilon}_{norm}$: Nominal (engineering) strain
